# E3 ubiquitin ligase FBXW11-mediated downregulation of S100A11 promotes sensitivity to PARP inhibitor in ovarian cancer

**DOI:** 10.1016/j.jpha.2025.101246

**Published:** 2025-02-27

**Authors:** Ligang Chen, Mingyi Wang, Yunge Gao, Yanhong Lv, Lianghao Zhai, Jian Dong, Yan Chen, Xia Li, Xin Guo, Biliang Chen, Yi Ru, Xiaohui Lv

**Affiliations:** a Department of Gynecology and Obstetrics, Xijing Hospital, Air Force Medical University, Xi'an, 710032, China; b Department of Gynecology and Obstetrics, The General Hospital of Western Theater Command PLA, Chengdu, 610036, China; c Department of Biochemistry and Molecular Biology, Air Force Medical University, Xi'an, 710032, China; d Department of Endoscopic Surgery, Chinese People's Liberation Army 986th Hospital, Air Force Medical University, Xi'an, 710054, China

**Keywords:** FBXW11, S100A11, PARPi resistance, Ovarian cancer

## Abstract

Resistance to poly adenosine diphosphate (ADP)-ribose polymerase inhibitor (PARPi) presents a considerable obstacle in the treatment of ovarian cancer. F-box and tryptophan-aspartic (WD) repeat domain containing 11 (FBXW11) modulates the ubiquitination of growth-and invasion-related factors in lung cancer, colorectal cancer, and osteosarcoma. The function of FBXW11 in PARPi therapy is still ambiguous. In this study, RNA sequencing (RNA-seq) showed that *FBXW11* expression was raised in ovarian cancer cells that had been treated with PARPi. FBXW11 was abnormally expressed at low levels in high-grade serous ovarian cancer (HGSOC) tissues, and low levels of FBXW11 were associated with shorter overall survival (OS) and progression-free survival (PFS) in HGSOC patients. Overexpressing FBXW11 made ovarian cancer more sensitive to PARPi, while knocking down FBXW11 made it less sensitive. The four-dimensional (4D) label-free quantitative proteomic analysis revealed that FBXW11 targeted S100 calcium binding protein A11 (S100A11) and promoted its degradation through ubiquitination. The increased degradation of S100A11 led to less efficient DNA damage repair, which in turn contributed to increased PARPi-induced DNA damage. The role of FBXW11 in promoting PARPi sensitivity was also confirmed in xenograft mouse models. In summary, our study confirms that FBXW11 promotes the susceptibility of ovarian cancer cells to PARPi via affecting S100A11-mediated DNA damage repair.

## Highlights

• FBXW11 expression increases in ovarian cancer cells treated with olaparib.
• LOW FBXW11 expression shows poor prognosis and higher tolerance to PARPi in HGSOC.
• FBXW11 increased olaparib-induced DNA damage by promoting S100A11 degradation.
• FBXW11 has great potential in the treatment of PARPi in ovarian cancer.

## Graphical abstract

**Figure:**
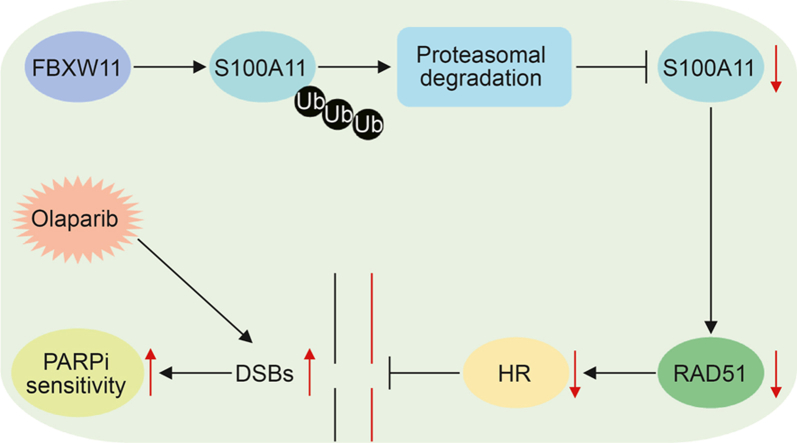

## 1 Introduction

Ovarian cancer is the gynecological neoplasm with the greatest fatality rate [1,2]. Owing to the absence of characteristic clinical symptoms of early ovarian cancer, 75% of impacted women receive a diagnosis of advanced-stage illness, and approximately 70% of patients succumb within five years [3]. The conventional standard therapy for ovarian cancer is cytoreductive operation in conjunction with platinum-and taxane-based therapy [4,5]. With improvements in surgical techniques and progress in neoadjuvant chemotherapy, patient prognosis has greatly improved; however, the majority of patients will experience a recurrence within five years and ultimately acquire medication resistance [6,7]. In recent years, various poly adenosine diphosphate (ADP)-ribose polymerase inhibitor (PARPi), such as olaparib, have received approval for treatment maintenance in patients with advanced ovarian cancer. Despite PARPi markedly extending progression-free survival (PFS) in individuals with ovarian cancer, clinical investigations indicate that 35% of patients exhibit insensitivity to olaparib, and a growing proportion of initially sensitive individuals develop resistance to PARPi [[8], [9], [10]]. Consequently, to allow a greater number of patients to benefit from PARP inhibitors, it is essential to investigate the reasons for PARPi resistance and devise techniques to counteract this resistance.

F-box and tryptophan-aspartic (WD) repeat domain containing 11 (FBXW11) participates in numerous essential biological processes, encompassing proliferation, differentiation, development, and metabolism, through its ability to target a broad range of substrates for degradation [11,12]. FBXW11 performs distinct functions in various tumor types, acting as either an oncogene or tumor suppressor. A study by Tan et al. [13] revealed that FBXW11 upregulation promotes nuclear factor-kappaB (NF-κB) pathway activation in pancreatic ductal adenocarcinoma, thereby facilitating tumor metastasis and impeding tumor cell apoptosis. According to Wang et al. [14,15], FBXW11 is upregulated in lymphocytic leukemia and facilitates leukemia cell proliferation by turning on the NF-κB pathways. Yao et al. [16] discovered FBXW11 augments stem cell-like characteristics and facilitates liver metastasis of colorectal cancer by regulating hypermethylated in cancer-1(HIC1)-mediated sirtuin-1 (SIRT1) expression. The upregulation of FBXW11, an immediate target of miR-182, in cervical cancer enhances tumor proliferation [17]. Conversely, in non-small cell lung cancer, FBXW11 is downregulated as an immediate target of miR-182, similarly promoting tumor proliferation and invasion [18]. Moreover, miR-221 inhibits the Wnt signaling pathway by directly targeting FBXW11, thereby facilitating osteosarcoma cell proliferation and suppressing apoptosis [19]. Chen et al. [20] reported significant downregulation of FBXW11 expression in human chondrosarcoma, and this downregulation was significantly associated with high-grade chondrosarcoma and a poor prognosis. However, FBXW11 expression in high-grade serous ovarian cancer (HGSOC) and its relationship with PARPi sensitivity have not been investigated.

S100 calcium binding protein A11 (S100A11) is associated with multiple types of cancer and plays unique roles in specific tumor subtypes. In the majority of malignancies, elevated S100A11 expression levels are closely associated with tumor promotion and progression [21,22]. Zagryazhskaya et al. [23] demonstrated that the downregulation of S100A11 using RNA interference markedly increases the vulnerability of non-small cell lung cancer cells to oxaliplatin, 5-fluorouracil, and cisplatin. Additionally, Cui et al. [24] shown that the suppression of S100A11 markedly enhances the vulnerability of gastric cancer cells to 5-fluorouracil and cisplatin. In ovarian cancer, S100A11 facilitates carcinoma growth and dissemination, and high S100A11 expression correlates with worse PFS and overall survival (OS) [[25], [26], [27]]. However, there is a lack of relevant studies on the association between S100A11 and PARPi sensitivity in ovarian cancer. Foertsch et al. [28] reported that S100A11 promotes DNA damage repair by enhancing the *Saccharomyces cerevisiae* (*S. cerevisiae*) homolog of *Escherichia coli* (*E. coli*) RecA (RAD51) strand exchange activity and recruiting RAD51 to sites of DNA damage. Since PARPi is categorized as DNA damage drugs, it is imperative to investigate the correlation between S100A11 and PARPi sensitivity.

In this study, we initially elucidated the expression levels of FBXW11 in patients diagnosed with HGSOC and its correlation with patient prognosis. We subsequently demonstrated that FBXW11 enhances the vulnerability of ovarian cancer cells to PARPi through the regulation of S100A11-mediated DNA damage repair processes. These findings present an innovative therapeutic approach to surmount PARPi resistance in ovarian cancer.

## 2 Materials and methods

### 2.1 RNA sequencing (RNA-seq)

A2780, SKOV3, and OV90 cells were grown in media supplemented with olaparib at doses of 30, 50, and 25 μM for 72 h. The subsequent step involved conducting RNA-seq on the three cell types both before and after olaparib treatment, with three replicate samples per group, yielding an overall sum of 18 samples. Total RNA was isolated from these 18 samples utilizing TRIzol (15596018; Thermo Scientific Inc., Waltham, MA, USA) following the directions given by the manufacturer. Samples exhibiting concentrations exceeding 50 ng/μL, RNA integrity number values surpassing 7.0, and total RNA greater than 1 μg were utilized for subsequent investigations. The samples were sequenced on an Illumina NovaSeq 6,000 system (LC Bio Technology, Hangzhou, China) according to the manufacturer's guidelines. Detailed procedures can be found in the Supplementary data.

### 2.2 Bioinformatics analysis

The expression quantities of FBXW11 across various categories of tumors and their associated normal tissues were acquired from the UALCAN (http://ualcan.path.uab.edu/) and TNMplot (https://tnmplot.com/analysis/) websites.

### 2.3 Patients and sample collection

A total of 32 fresh tissue samples of HGSOC and 16 fresh tissue samples of normal ovarian tissue were acquired from the Department of Obstetrics and Gynecology, Xijing Hospital, Air Force Medical University (Xi'an, China), from October 2022 to June 2023 to evaluate the protein and messenger RNA (mRNA) levels of FBXW11. Additionally, a total of 169 paraffin cases of HGSOC and 35 normal ovary samples collected at Pathology Department of Xijing Hospital between January 2017 and December 2018 were used to assess the expression of FBXW11 and S100A11 through IHC. The HGSOC tissues were sourced from patients diagnosed with primary ovarian cancer without previous surgical or chemotherapy history, whereas the normal ovarian tissues were acquired from individuals having adnexectomy for benign illnesses. The study obtained approval from the Ethics Committee of Xijing Hospital (Approval No.: KY20193023), and all sample collection and human experimentation techniques complied with the rules established by this committee.

### 2.4 Cell lines and cell culture

A2780, SKOV3, OV90, UWB1.289, and IOSE80 cells were acquired from Shanghai Cellular Biology Center (Shanghai, China). The authenticity of all cell lines was confirmed based on the short tandem repeat (STR) profile, and thorough testing for mycoplasma contamination was conducted to ensure its absence. A2780, SKOV3, OV90, UWB1.289, and IOSE80 cell lines were grown at 37 °C in a 5% CO_2_ environment, utilizing Dulbecco's modified Eagle medium‌ (DMEM) or Roswell Park Memorial Institute-1640 (RPMI-1640) media (Gibco, Waltham, MA, USA) supplied with 10% fetal bovine serum 1% penicillin, and 1% streptomycin. The FBXW11 small interfering RNA (siRNA) was developed and produced by GenePharma (Shanghai, China). FBXW11-siRNA was introduced into A2780, SKOV3, and OV90 cells to obtain FBXW11 transient knockdown cell lines. For the generation of FBXW11 stable knockdown and overexpression in ovarian cancer cells, SKOV3, A2780, OV90, and UWB1.289 cells were seeded onto six-well plates with 40%–50% confluence. Cells were then transfected with lentivirus carrying FBXW11 short hairpin RNA (shRNA) or HanBio lentivirus (HBLV) FBXW11 (Hanbio Biotechnology Co., Ltd., Shanghai, China) respectively. After 72 h of transduction, cells were subjected to puromycin selection (2 mg/mL). GENERAL BIOL (Anhui, China) designed and provided S100A11 overexpression plasmids. S100A11 overexpression plasmids was introduced into A2780 and UWB1.289 cells to obtain S100A11 transient overexpression cell lines.

### 2.5 Western blot analysis and antibodies

The cells underwent treatment with RIPA lysis buffer (P0013B; Beyotime Biotechnology, Shanghai, China) containing phosphatase inhibitors (P1049-2; Beyotime Biotechnology) and broad-spectrum protease inhibitors (P1049-1; Beyotime Biotechnology), then the protein lysates were collected. The entire cell lysate, at a uniform mass of 50 μg, was fractionated using a 10% sodium dodecyl sulfate-polyacrylamide gel electrophoresis (SDS-PAGE) and subsequently transferred to nitrocellulose (NC) blotting membranes. After incubation with the corresponding primary antibody, NC membranes were subsequently incubated with mouse or rabbit secondary antibodies. Enhanced chemiluminescence liquid (ECL) tests were employed to visualize the blots (P0018AM; Beyotime Biotechnology). The results were analyzed utilizing the ImageJ software (V1.8.0.112; National Institutes of Health, Bethesda, MD, USA). The antibodies utilized in this study, along with their respective dilution ratios, are detailed below. Antibodies were obtained from Proteintech (Wuhan, China) as follows: anti-FBXW11 (1:5000; 68090-1-Ig), anti-glyceraldehyde-3-phosphate dehydrogenase (GAPDH) (1:2000; 60004-1-Ig), and anti-S100A11 (1:2000; 60024-1-Ig). Antibodies were obtained from Cell Signaling Technology (Beverly, MA, USA) as follows: anti-caspase-3 (1:1000; 14220T), anti-PARP (1:1000; 9532T), anti-cleaved caspase-3 (1:1000; 9661T), anti-cleaved PARP (1:1000; 5625T), anti-mouse-IgG horseradish peroxidase (HRP)-linked (1:3000; 7076P2), and anti-rabbit IgG HRP-linked (1:3000; 7074P2). Anti-γH2AX (1:1000; ab22551) was obtained from Abcam (Cambridge, UK).

### 2.6 Immunohistochemistry (IHC)

IHC staining was conducted on paraffin slices following the manufacturer's technique. The sections were exposed to heat treatment at 60 °C for 30 min and then underwent xylene washing to remove any waxy residues. Subsequent to this phase, the samples were rehydrated with various quantities of ethanol. The antigens were recovered by autoclaving the tissue slices in 10 mM citrate buffer at pH 6.0 for 5 min. Subsequent to cooling to room temperature, slices were subjected to 3% H_2_O_2_ for 20 min to inhibit endogenous peroxidase activity, followed by a 60-min incubation in goat serum to diminish nonspecific interactions. Thereafter, samples were treated overnight at 4 °C with rabbit monoclonal antibodies targeting FBXW11(1:200; PA5-99044; Invitrogen, Carlsbad, CA, USA) and S100A11 (1:200; 10237-1-AP; Proteintech). Subsequent to incubation with the appropriate secondary antibodies, the sections underwent diamine benzidine staining, followed by counterstaining with hematoxylin. The degree of staining was assessed according to the percentage of tumor cells displaying positive staining: 1 (0%–25%), 2 (26%–50%), 3 (51%–75%), and 4 (76%–100%). The staining intensity was assessed on a scale of 0 (negative), 1 (weakly positive), 2 (moderately positive), and 3 (highly positive). The IHC score is derived by multiplying two scores, with the final result for each sample being the average of the scores from two replicate samples. A high expression level was characterized as an IHC score ≥ 3, while a low expression level was defined as an IHC score < 3.

### 2.7 Quantitative real-time reverse transcription polymerase chain reaction (qRT-PCR)

Following the directions given by the manufacturer, TRIzol reagent (15596018CN; Invitrogen) was used to isolate total RNA from cells and tissues. The RNA underwent reverse transcription to produce complementary DNA (cDNA) utilizing the qPCR Kit (11141ES60; Yeasen, Shanghai, China). Cycling was initiated using the qRT-PCR premixed solution after addition of the corresponding primers (11201ES08; Yeasen). Actin functioned as an internal reference. The relative quantification of qRT-PCR data was conducted utilizing the 2^−ΔΔ*C*^_*T*_ approach. The PCR primers were utilized as detailed below: FBXW11 forward: 5′-CTTCTGCGACCGACATCACTTTA-3′, FBXW11 reverse: 5′-GCCCATTGAGAGTACGAACAAATT-3′; S100A11 forward: 5′-GCATTGAGTCCCTGATTGCT-3′, S100A11 reverse: 5′-ATCTAGCTGCCCGTCACAGT-3′; and actin forward: 5′- GGCTGTATTCCCCTCCATCG -3′, actin reverse: 5′-CCAGTTGGTAACAATGCCATGT-3′.

### 2.8 Cell counting Kit-8 (CCK-8) assay

Treated cells were seeded in 96-well plates and cultured with 200 μL of culture media, followed by the addition of 20 μL of CCK-8 (C0037; Beyotime Biotechnology) to each well, and incubated for 3 h at 37 °C. The Varioskan Flash microplate reader (Thermo Scientific Inc.) was used to measure the absorbance at a wavelength of 450 nm.

### 2.9 Colony formation assay

Six well plates were inoculated with 700 cells per well. The appropriate concentration of olaparib was added to the medium and incubated for a period of 7–14 days. Crystal violet was employed to stain the colonies after they were fixated with methanol. The quantity of colonies exceeding 50 cells was measured utilizing ImageJ software program (V1.8.0.112; National Institutes of Health). In ImageJ software, Analyze-Analyze particle-size (pixel × pixel) was set from 100 to 1,000, so that clones with more than 50 cells would be counted.

### 2.10 5-Ethynyl-20-deoxyuridine (EdU) incorporation assay

The cells that were treated were seeded at a density of 5 × 10^4^ cells per well in a 12-well plate and subsequently assessed using the Cell Proliferation Kit (C0085S; Beyotime Biotechnology), adhering to the directions provided by the manufacturer. The EdU-positive cells were examined utilizing fluorescence microscopy.

### 2.11 Apoptosis detection

The treated cells were cleaned with phosphate-buffered saline (PBS), exposed to trypsin digestion, and subsequently rinsed with cold PBS. The induction of apoptosis was assessed using a flow cytometer (Becton, Dickinson and Company, Franklin Lakes, NJ, USA) along with the Annexin V-PE/7-AAD Apoptosis Detection Kit (40310ES50, Yeasen). Terminal deoxynucleotidyl transferase (TdT) dUTP nick-end labeling (TUNEL) assay was conducted to detect DNA fragmentation following the directions given by the manufacturer (40306ES50; Yeasen).

### 2.12 Four-dimensional (4D) label-free quantitative proteomic analysis

A2780 cells in the stable FBXW11 overexpression group and control group were washed with PBS pre-cooled at 4 °C. Subsequently, adherent cells were collected from the dishes using a cell scraper, and the detached cells were transferred into a pre-cooled centrifuge tube for subsequent centrifugation and removal of the supernatant. The cellular precipitation was promptly frozen with liquid nitrogen and subsequently transported under dry ice to GeneChem (Shanghai, China) for 4D label-free quantitative proteomic analysis. Each group consisted of three replicate samples, resulting in a total of six samples. Comprehensive experimental protocols are included in the Supplementary data.

### 2.13 Co-immunoprecipitation (Co-IP) and *in vivo* ubiquitination assays

The IP and IgG antibodies of the identical species were mixed with 20 μL of protein A/G magnetic beads (P2108; Beyotime Biotechnology) overnight at 4 °C, following the manufacturer's guidelines. Cells were broken down with lysis buffer (P0013; Beyotime Biotechnology) and then centrifugation for 15 min at low temperature. Cell supernatants (1,000 μg) were treated for an entire night at 4 °C with A/G magnetic beads attached to IP or IgG antibodies. Subsequently, the antigens were eluted and subjected to Western blot analysis for antigen detection. Anti-S100A11 (rabbit; 4 μg; 10237-1-AP; Proteintech), anti-FBXW11 (rabbit; 4 μg; 13149-1-AP; Proteintech), and anti-IgG (rabbit; 4 μg; 30000-0-AP; Proteintech) were used for IP. Anti-ubiquitin (mouse; 1:1000; 3936T; Cell Signaling Technology), anti-S100A11 (mouse; 1:2000; 60024-1-Ig; Proteintech), and anti-FBXW11 (mouse; 1:5000; 68090-1-Ig; Proteintech) were used for immunoblotting (IB). For the *in vivo* ubiquitination assay, A2780 and UWB1.289 cells underwent treatment with 20 μM MG132 for 4 h, after which the cell lysates were incubated with magnetic beads coupled to the S100A11 antibody, allowing for the detection of S100A11 ubiquitination in the anti-S100A11 pull-down precipitation.

### 2.14 Immunofluorescent staining and confocal microscopy

Confocal dishes with a diameter of 20 mm were used to plate the cells. When the cell density reached 60 %–80%, the cells were stained with 4% paraformaldehyde for 15 min. Following PBS cleaning, cells were exposed to 0.5% Triton X-100 for 5 min. After being blocked for 30 min, the cells were incubated with primary antibodies overnight. Following three rounds of phosphate-buffered saline with tween 20 (PBST) washes, the relevant secondary antibodies were applied and incubated for 2 h. The 4′,6-diamidino-2-phenylindole (DAPI) (G1012; Servicebio, Wuhan, China) was used to counterstain the nuclei for 5 min at 37 °C. The antifading solution (P0126; Beyotime Biotechnology) was administered dropwise and subsequently observed and captured using a Nikon A1+ laser confocal microscope (Nikon Corporation, Tokyo, Japan). The antibodies were as follows: anti-S100A11 (1:200; 60024-1-Ig; Proteintech), anti-FBXW11 (1:200; 13149-1-AP; Proteintech), anti-γH2AX (1:200; ab22551; Abcam), anti-Mouse IgG (1:200; ab150107; Abcam), and anti-Rabbit IgG (1:200; ab150075; Abcam).

### 2.15 DNA damage repair assays

When DNA damage occurs, H2A histone family member X (H2AX) located at the site of DNA damage is phosphorylated at the 139th serine residue, and this phosphorylated H2AX protein is referred to as γH2AX. The recruitment and localization of DNA repair proteins start with γH2AX, which is dephosphorylated after DNA repair. Therefore, the dynamic detection of γH2AX level by Western blot and immunofluorescence can reflect the extent of DNA damage and repair efficiency in cells.

### 2.16 Xenograft tumor models

To minimize the influence of extraneous variables, six-week-old female Balb/c nude mice were reared in a pathogen-free environment at the Animal Experimental Center of Air Force Military Medical University. Each mouse test was carried out in adhering to the animal rules sanctioned by the Medical Ethics Committee of the Xijing Hosptial, Air Force Military Medical University. Each mouse was subcutaneously injected with 5 × 10^6^ SKOV3 cells transfected with HBLV or HBLV FBXW11 in the left armpit. The olaparib compound was solubilized in dimethyl sulfoxide (DMSO) and diluted with PBS prior to injection. A vehicle control consisting of 10% DMSO in PBS was employed. When the tumor volumes attained roughly 50 mm^3^, the mice were distributed at random into four groups (HBLV, HBLV FBXW11, HBLV + olaparib, and HBLV FBXW11 + olaparib) and administered an intraperitoneal dose of olaparib (50 mg/kg) or PBS daily. Animal experiments were approved by the Medical Ethics Committee of Xijing Hospital, Air Force Medical University (Xi'an, China) (Approval No.: KY20193023). All experimental procedures involving animals were conducted in strict accordance with the approved guidelines and protocols.

### 2.17 Experiment reagents

Olaparib (AZD2281, HY-10162), cycloheximide (CHX) (HY-12320), and MG132 (HY-13259) were acquired from MedChemExpress (New York, NJ, USA).

### 2.18 Statistical analysis

SPSS27 software was used for statistical analysis. Student's *t*-test was utilized to analyze two independent groups. Three or more groups were subjected to the analysis of variance (ANOVA) test. The data are shown as mean ± standard error of the mean (SEM), unless otherwise specified. Spearman test was employed to assess the connection between FBXW11 and S100A11. The relationship between PFS, OS, and FBXW11 levels was analyzed employing Kaplan-Meier survival analysis. *P*-values < 0.05 were deemed to indicate statistically significant differences (^∗^*P* < 0.05 and ^∗∗^*P* < 0.01).

## 3 Results

### 3.1 *FBXW11* expression is upregulated in ovarian cancer cells upon treatment with olaparib

**Fig. 1:**
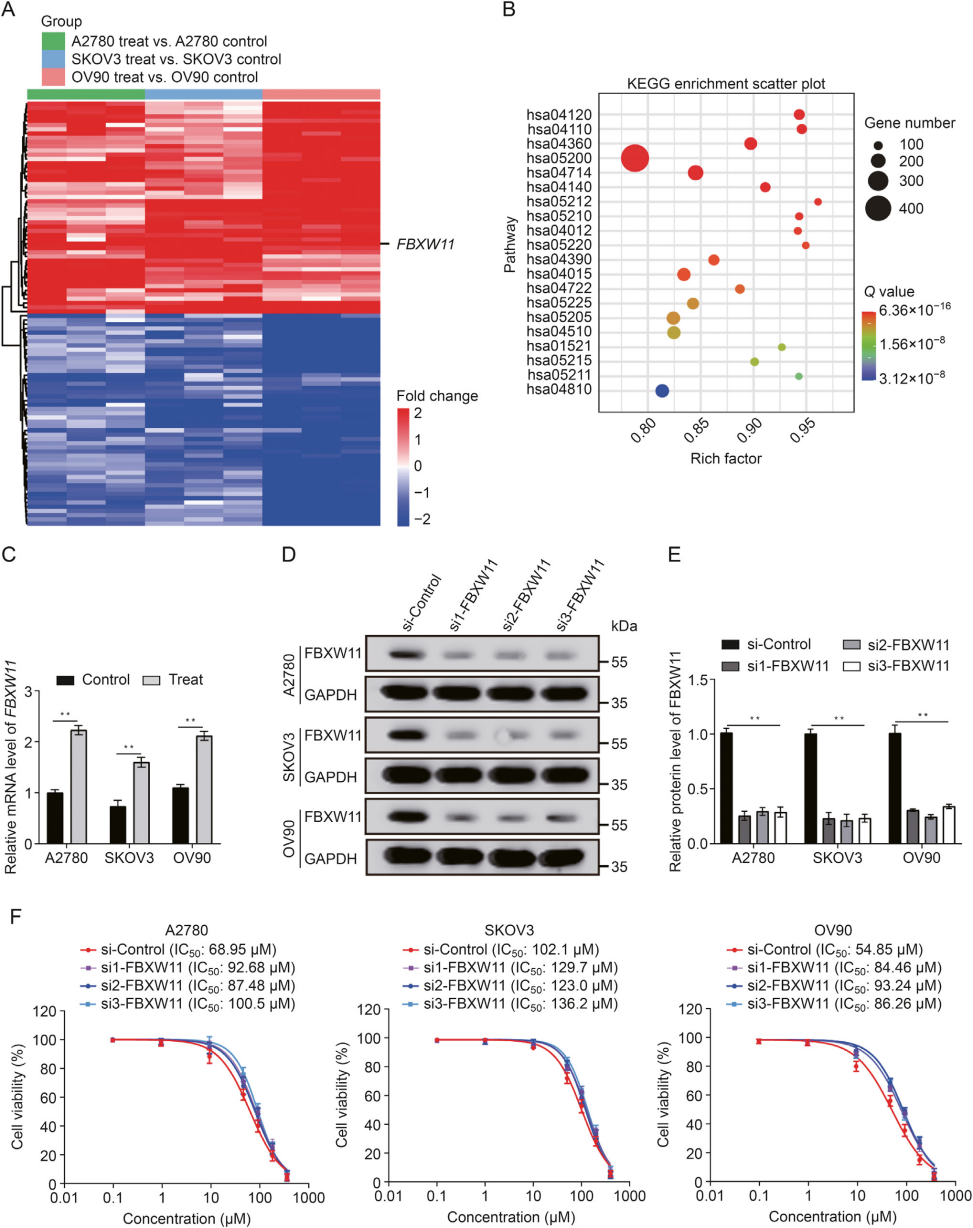
F-box and tryptophan-aspartic (WD) repeat domain containing 11 (*FBXW11*) is upregulated in ovarian cancer cells subjected to olaparib treatment. (A) RNA sequencing (RNA-seq) was performed to detect genetic differences in three kinds of cell lines treated with olaparib (A2780, 60 μM; SKOV3 100 μM; and OV90 50 μM) for 72 h. In the 18 cell samples from the three cell lines subjected to olaparib therapy, 989 genes were found to be upregulated and 1030 genes downregulated. The heat map shows the 50 upregulated and 50 downregulated genes with the largest fold change. (B) The results of Kyoto Encyclopedia of Genes and Genomes (KEGG) enrichment analysis for the gene expression differences across the three cell lines. (C) *FBXW11* messenger RNA (mRNA) levels before and after 72 h of olaparib treatment were determined by quantitative real-time reverse transcription polymerase chain reaction (qRT-PCR) (A2780, 60 μM; SKOV3 100 μM; and OV90 50 μM). (D, E) Western blot demonstrated the expression of FBXW11 in small interfering (si)-FBXW11 and si-control (D) and quantification of FBXW11 protein levels (E). (F) The Cell Counting Kit-8 (CCK-8) assay was employed to ascertain the half maximal inhibitory concentration (IC_50_) values of the three ovarian cell types following 72 h of olaparib treatment. Fig. S1 displays the statistical analysis's findings. ^∗∗^*P* < 0.01. GAPDH: glyceraldehyde-3-phosphate dehydrogenase.

To investigate the genes involved in the cytotoxic effect of olaparib, we employed RNA-seq technology to assess A2780, SKOV3, and OV90 cell lines before and after olaparib treatment. Kyoto Encyclopedia of Genes and Genomes (KEGG) pathway enrichment research showed that ubiquitin-mediated protein degradation was highly enriched. Therefore, among the upregulated genes, *FBXW11*, the E3 ubiquitin ligase exhibiting the most significant fold change following olaparib treatment, was chosen for subsequent experimental investigations (Fig. 1A). The KEGG enrichment analysis results indicated that the ubiquitin-mediated proteolysis pathway (hsa04120) presented one of the highest degrees of enrichment (Fig. 1B). We confirmed the upregulation of *FBXW11* expression following olaparib treatment using qRT-PCR (Fig. 1C). To clarify whether the upregulation of *FBXW11* contributes to olaparib-mediated killing or enhances olaparib tolerance in ovarian cancer cells, we silenced the expression of FBXW11 in these three cell lines using siRNA (Figs. 1D and E). We then determined the half maximal inhibitory concentration (IC_50_) value by CCK-8 assay, and we found that after knockdown of FBXW11, the IC_50_ value of olaparib was increased compared with the control group in all three ovarian cancer cell lines (Figs. 1F and S1). Therefore, we hypothesized that olaparib increases its cytotoxicity by upregulating FBXW11 within ovarian carcinoma cells.

### 3.2 Low FBXW11 expression suggests a poor prognosis in HGSOC

**Fig. 2:**
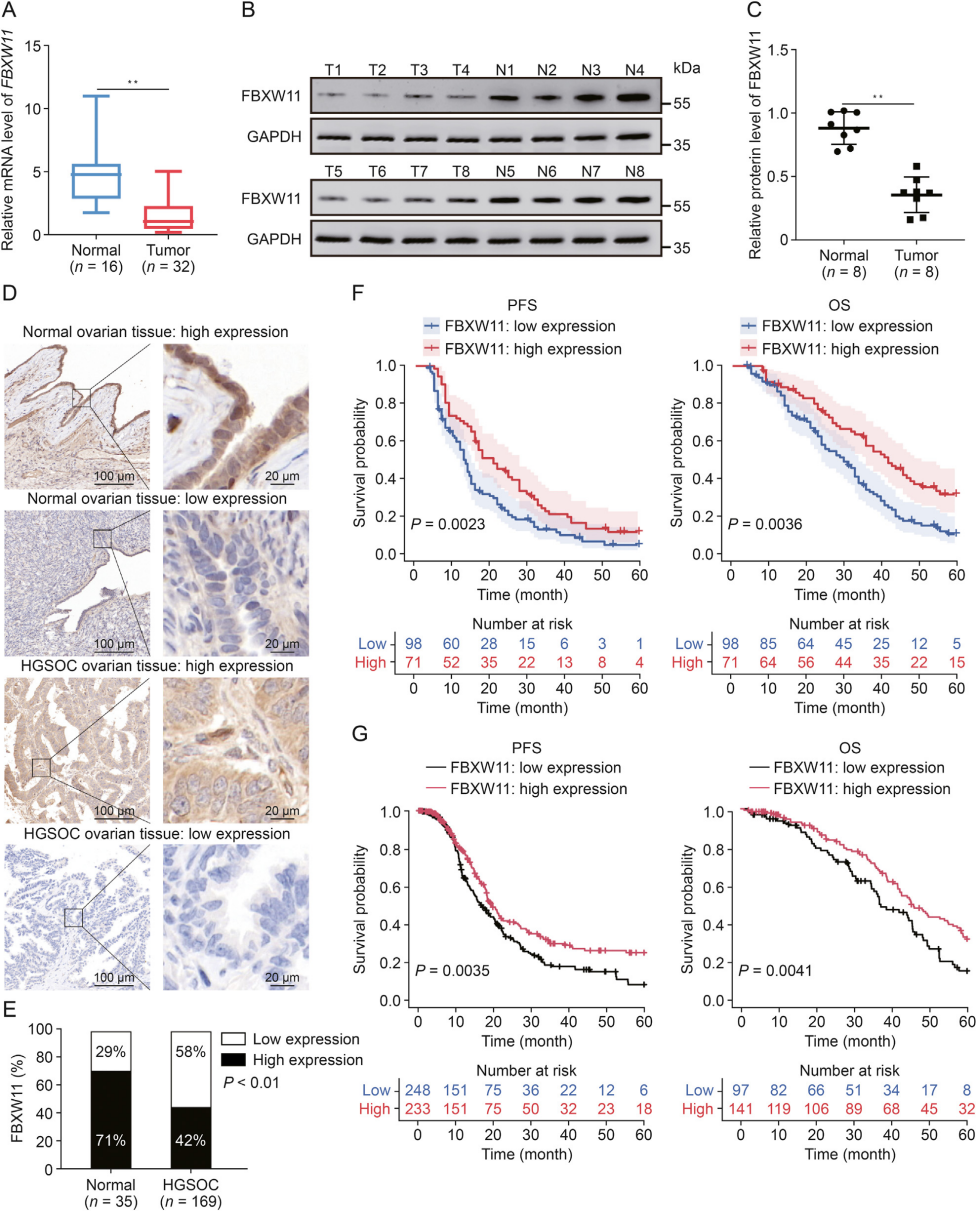
F-box and tryptophan-aspartic (WD) repeat domain containing 11 (FBXW11) is expressed at low levels and is associated with worse prognosis in high-grade serous ovarian cancer (HGSOC). (A) The transcript levels of *FBXW11* in fresh tissue samples were determined by quantitative real-time reverse transcription polymerase chain reaction (qRT-PCR). (B, C) The protein levels of FBXW11 in fresh tissue samples determined by Western blot (B) and quantification of FBXW11 protein levels (C). (D) Representative images of immunohistochemistry (IHC) staining of FBXW11 in normal ovarian tissue and HGSOC tissue. (E) The ratio of low and high expression of FBXW11 in the two tissues. (F) Kaplan-Meier curves for progression-free survival (PFS) and overall survival (OS) in HGSOC patients with low versus high FBXW11 expression levels. Data were obtained from clinical collections. (G) Kaplan-Meier curves for PFS and OS in HGSOC patients with low versus high FBXW11 expression levels. Data were obtained from The Cancer Genome Atlas (TCGA). ^∗∗^*P* < 0.01. T: tumor; N: normal; GAPDH: glyceraldehyde-3-phosphate dehydrogenase.

We initially examined FBXW11 expression in both ovarian cancer tissue and normal ovarian tissue. The Cancer Genome Atlas (TCGA) data indicated that FBXW11 is present at low levels in multiple cancers of human tissues, consisting of ovarian cancer (Fig. S2). Additionally, FBXW11 exhibited marked downregulation at both the mRNA and protein levels in HGSOC tissue (Figs. 2A−C). In order to evaluate the clinical value of FBXW11, we conducted IHC staining of HGSOC and normal ovarian tissues. Among the HGSOC tissue samples, 58% were classified as having low FBXW11 expression, whereas only 29% of the normal ovarian tissue samples were classified as such (Figs. 2D and E). Survival analysis indicated that the PFS and OS of patients exhibiting low FBXW11 expression were markedly inferior to those of patients with elevated FBXW11 expression, and the same results were obtained using data from the TCGA database (Figs. 2F and G). These results highlight the clinical importance of investigating the role of FBXW11 in the context of PARPi treatment of ovarian cancer.

### 3.3 FBXW11 promotes the sensitivity of ovarian cancer cells to olaparib

**Fig. 3:**
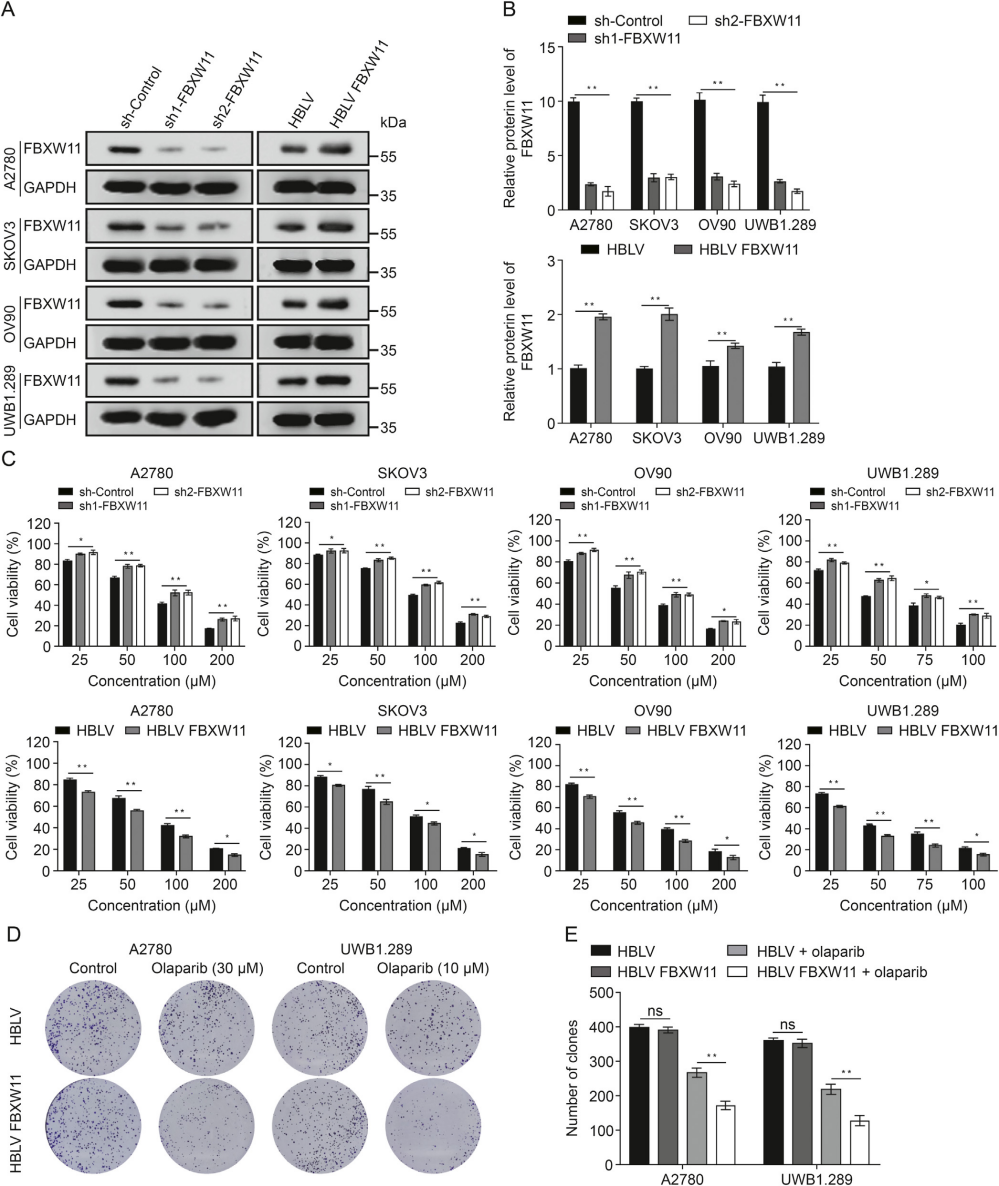
F-box and tryptophan-aspartic (WD) repeat domain containing 11 (FBXW11) augmented the inhibition of ovarian cancer cell proliferation by olaparib. (A, B) Western blot was employed to assess the knockdown and overexpression efficacy of FBXW11 in the four ovarian cancer cell lines (A) and quantification of FBXW11 protein levels (B). (C) The vitality of these four ovarian cancer cells subjected to olaparib treatment for 72 h was evaluated utilizing the Cell Counting Kit-8 (CCK-8) assay. (D, E) Colony formation test was utilized to measure the colony formation rate of cells subjected to olaparib treatment for 7–14 days (D) and measurement of the clone count (E). ^∗^*P* < 0.05 and ^∗∗^*P* < 0.01. ns: not significant. GAPDH: glyceraldehyde-3-phosphate dehydrogenase; sh: short hairpin; HBLV: HanBio lentivirus.

**Fig. 4:**
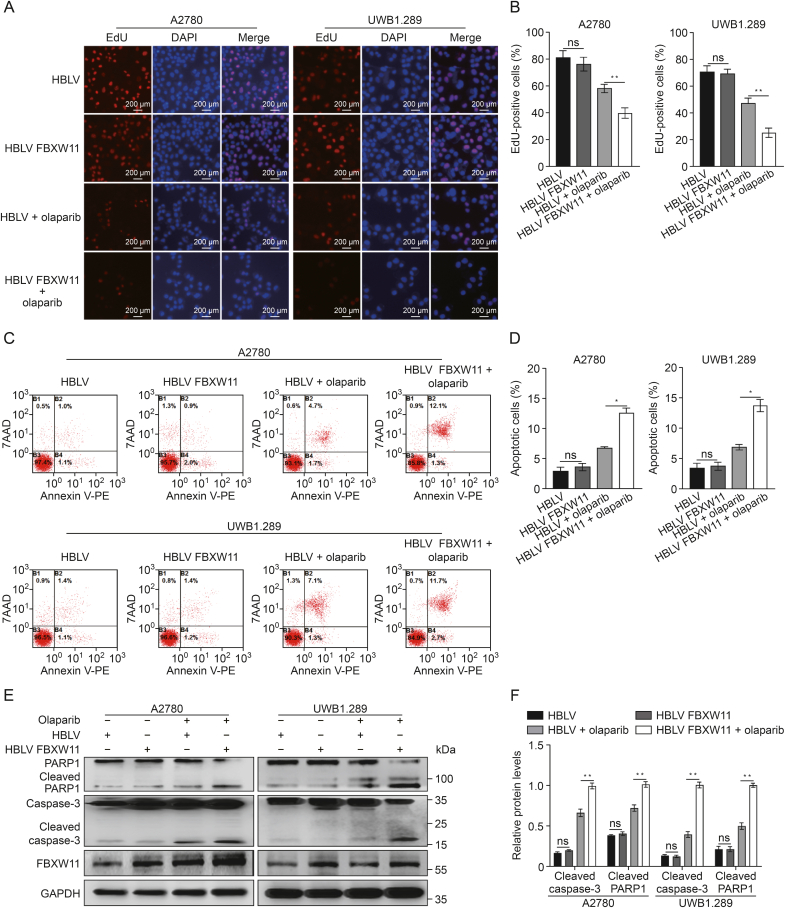
F-box and tryptophan-aspartic (WD) repeat domain containing 11 (FBXW11) enhances olaparib-induced apoptosis. (A, B) 5-Ethynyl-20-deoxyuridine (EdU) method was employed to evaluate cell proliferation in the HanBio lentivirus (HBLV) and HBLV FBXW11 groups. The drug-treatment groups underwent olaparib (A2780, 30 μM and UWB1.289, 10 μM) processing 72 h prior to testing (A) and quantification of the percentage of EdU-positive cells (B). (C, D) The HBLV and HBLV FBXW11 groups were tested for cell apoptosis using a flow cytometry technique. The drug-treatment groups underwent olaparib (A2780, 30 μM and UWB1.289, 10 μM) processing 72 h prior to testing (C) and quantification of the proportion of apoptotic cells (D). (E, F) The two ovarian cancer cell lines, transfected with HBLV and HBLV FBXW11, were subjected to olaparib treatment (A2780 at 30 μM and UWB1.289 at 10 μM) for 72 h. Levels of cleaved poly adenosine diphosphate (ADP)-ribose polymerase 1 (PARP1), cleaved caspase-3, and FBXW11 were measured using Western blotting (E) and quantification of protein levels (F). ^∗^*P* < 0.05 and ^∗∗^*P* < 0.01. ns: not significant. DAPI: 4′,6-diamidino-2-phenylindole; 7AAD:7-aminoactinomycin D; PE: phycoerythrin; GAPDH: glyceralde-hyde -3-phosphate dehydrogenase.

We chose one breast cancer susceptibility gene (BRCA) mutant ovarian cancer cell line (UWB1.289) and three BRCA wild-type (A2780, SKOV3, and OV90) to establish stable FBXW11-knockdown and FBXW11-overexpressing cell lines. At first, we examined the expression of FBXW11 in the above four ovarian cancer cell lines, as well as one normal ovarian cell line called IOSE80, using Western blotting and qRT-PCR (Fig. S3). Subsequently, lentiviral vector transfection was employed to introduce sh control, FBXW11 shRNA, HBLV, and HBLV FBXW11 plasmids into A2780, SKOV3, OV90, and UWB1.289 cell lines in order to create stable cellular models exhibiting either reduced or increased expression of FBXW11. Compared to the control cells, there was a decrease in FBXW11 expression observed in the cells transfected with FBXW11-specific shRNA, whereas it was upregulated in the HBLV FBXW11 cells in comparison to the HBLV cells (Figs. 3A and B). Olaparib was more effective against the four ovarian cancer cells when FBXW11 was upregulated. Conversely, FBXW11-knockdown cells exhibited reduced sensitivity to olaparib (Fig. 3C). In the absence of olaparib in the culture medium, overexpression of FBXW11 did not affect the colony formation ability. However, in the presence of olaparib, the colony formation ability of the FBXW11-overexpressing group was markedly inferior to that of the control group (Figs. 3D and E). Correspondingly, the EdU proliferation assay results showed a markedly greater inhibitory effect of olaparib on FBXW11-overexpressing ovarian cancer cells than on the corresponding control cells (Figs. 4A and B).

The flow cytometry-based apoptosis test results indicated that FBXW11 overexpression elevated the percentage of apoptotic ovarian cancer cells when exposed to olaparib (Figs. 4C and D). Moreover, FBXW11 overexpression significantly increased the concentrations of apoptosis-related proteins in olaparib-treated cells (Figs. 4E and F). These findings indicate that FBXW11 upregulation contributes to the cytotoxic effect of olaparib in ovarian cancer cells.

### 3.4 S100A11 is a substrate of FBXW11

**Fig. 5:**
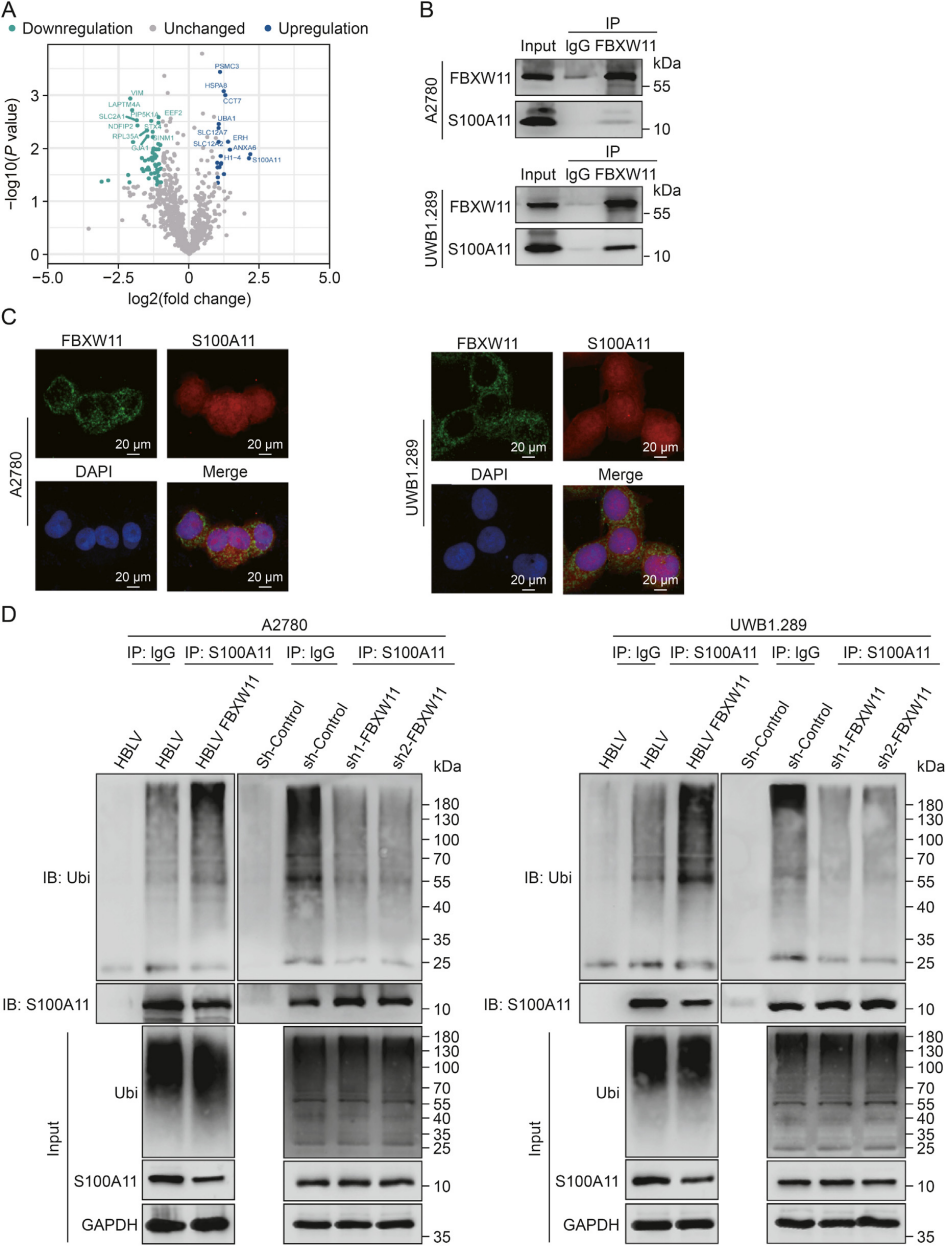
F-box and tryptophan-aspartic (WD) repeat domain containing 11 (FBXW11) specifically targets S100 calcium binding protein A11 (S100A11). (A) The volcano plot was drawn by the expression difference fold and *P* value of ubiquitination sites between HanBio lentivirus (HBLV) FBXW11 group and HBLV group in A2780 cells. (B) The intrinsic relationship between FBXW11 and S100A11 in ovarian cancer cells was validated via co-immunoprecipitation (Co-IP). (C) Immunofluorescence confocal analysis revealed the co-localization of FBXW11 and S100A11 within ovarian cancer cells. (D) The cells were subjected to a 4 h treatment with 20 μM MG132, and then the magnetic beads coated with the S100A11 antibody were utilized to incubate the cell lysates, and S100A11 ubiquitination was detected in anti-S100A11 pull-down precipitation. The two HBLV samples in the upper panel are the sample immunoprecipitated with IgG antibody on the left and the sample immunoprecipitated with S100A11 antibody on the right. DAPI: 4′,6-diamidino-2-phenylindole; sh: short hairpin; IB: immunoblotting; Ubi: ubiquitin; GAPDH: glyceraldehyde-3-phosphate dehydrogenase.

To elucidate the specific substrates targeted by FBXW11, we performed 4D label-free quantitative proteomic analysis of ubiquitination (performed by GeneChem). Notably, S100A11 showed a marked increase in the number of ubiquitinated sites after FBXW11 overexpression (Fig. 5A). To investigate the interaction between FBXW11 and S100A11, we obtained cell lysates from A2780 and UWB1.289 cells and performed Co-IP analysis with anti-IgG and anti-FBXW11 antibodies. S100A11 was distinctly identified in the precipitate isolated by anti-FBXW11 (Fig. 5B). Similarly, FBXW11 and S100A11 were found to colocalize in the cytoplasm and nucleus of A2780 and UWB1.289 cells using dual immunofluorescence labeling (Fig. 5C). Furthermore, we examined the ubiquitin levels in precipitates pulled down with an anti-FBXW11 antibody or anti-IgG as a control. The results revealed that the level of S100A11 ubiquitination increased following FBXW11 overexpression, but they diminished after FBXW11 knockdown (Fig. 5D). These results suggest that FBXW11 targets S100A11 and promotes its ubiquitination.

### 3.5 FBXW11 reduces the stability of S100A11

**Fig. 6:**
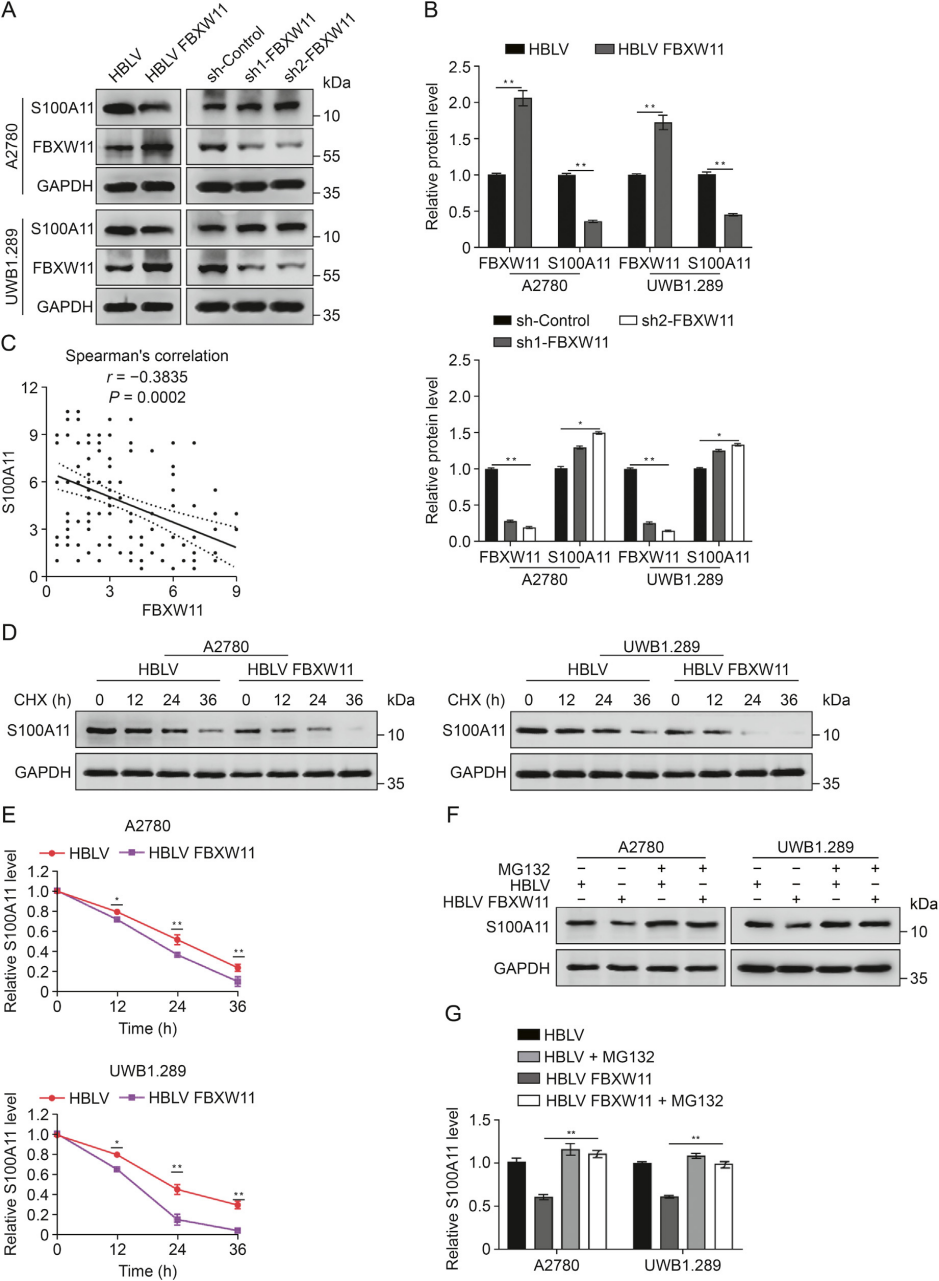
Effect of F-box and tryptophan-aspartic (WD) repeat domain containing 11 (FBXW11) on the stability of S100 calcium binding protein A11 (S100A11). (A, B) FBXW11 and S100A11 protein levels were assessed using Western blotting (A) and the relative levels of FBXW11 and S100A11 (B). (C) Correlation analysis between FBXW11 and S100A11 in 169 clinical tissue samples. (D, E) Transfected HanBio lentivirus (HBLV) and HBLV FBXW11 cells underwent treatment with 50 μM of cycloheximide (CHX). Protein expression of S100A11 was assessed using Western blotting after treatment for 0, 12, 24, and 36 h (D) and quantification of S100A11 relative protein levels (E). (F, G) S100A11 protein levels were measured by Western blotting following treatment of the cells with 20 μM MG132 for 8 h (F) and quantification of S100A11 protein levels (G). ^∗^*P* < 0.05 and ^∗∗^*P* < 0.01. sh: short hairpin; GAPDH: glyceraldehyde-3-phosphate dehydrogenase.

Next, we investigated whether the association of S100A11 with FBXW11 promotes ovarian cancer cell sensitivity to olaparib. The qRT-PCR results revealed no discernible disparity in *S100A11* expression at the transcriptional level between the HBLV group and the HBLV FBXW11 group (Fig. S4). Yet, S100A11 protein expression was markedly diminished in cells overexpressing FBXW11, whereas it was elevated in cells with FBXW11 knockdown (Figs. 6A and B), indicating a potential correlation between FBXW11 and S100A11 levels in ovarian cancer cells. In addition, we analyzed the correlation between FBXW11 and S100A11 by IHC analysis of the 169 clinical tissue samples mentioned above. The findings demonstrated a notable inverse association between FBXW11 and S100A11 (Fig. 6C). To explore whether the decrease in S100A11 levels following FBXW11 overexpression is due to increased protein degradation, we conducted CHX chase experiments. The findings indicated that FBXW11 overexpression expedited the degradation of S100A11 in ovarian cancer cells (Figs. 6D and E). We subsequently examined S100A11 expression in the FBXW11-overexpressing group and the control group after MG132 therapy. MG132 therapy markedly elevated the levels of S100A11 (Figs. 6F and G). In conclusion, FBXW11 accelerates the degradation of S100A11 through ubiquitination, leading to decreased protein levels.

### 3.6 FBXW11 enhances olaparib-induced DNA damage by facilitating the degradation of S100A11

**Fig. 7:**
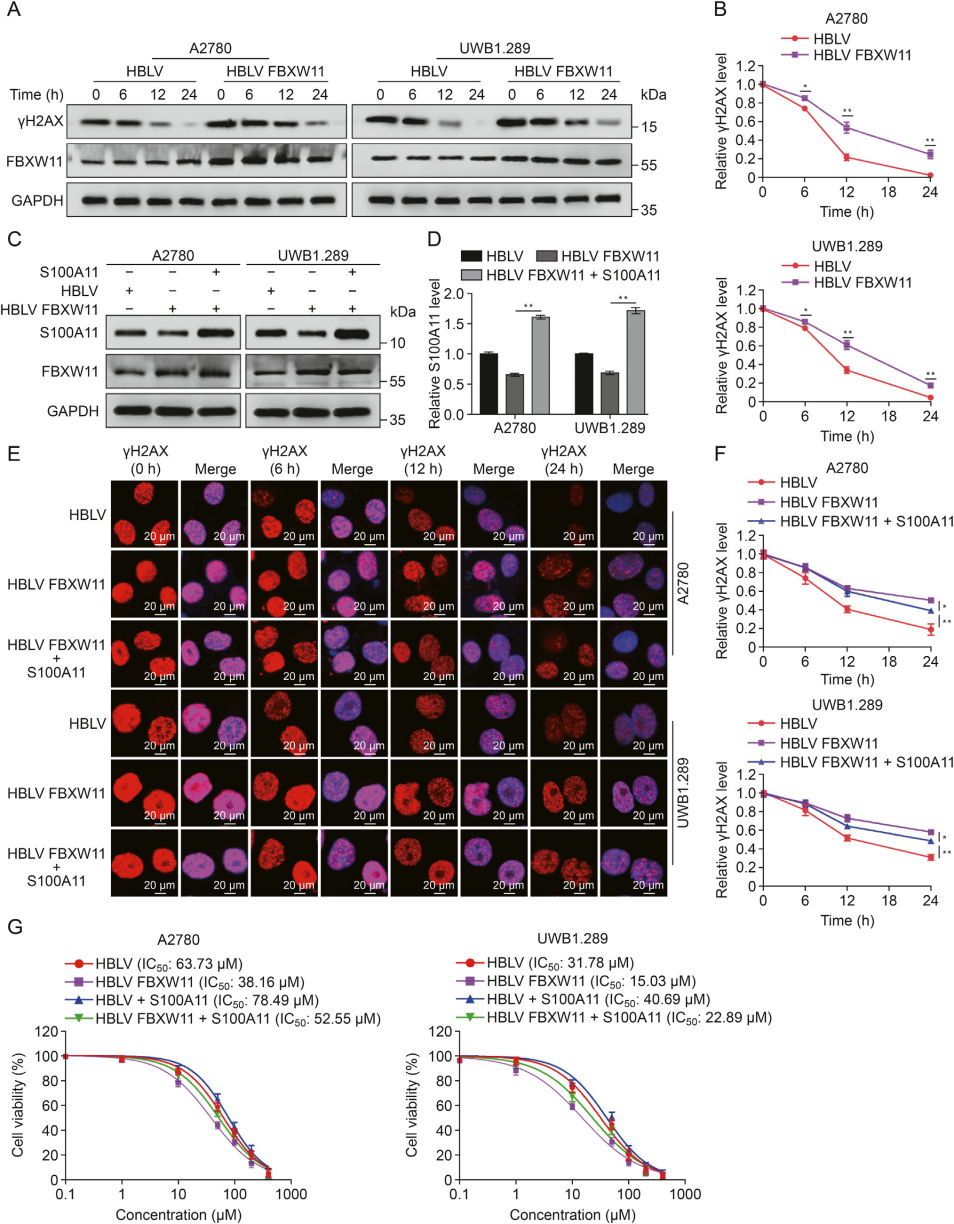
F-box and tryptophan-aspartic (WD) repeat domain containing 11 (FBXW11) enhances olaparib-induced DNA damage through S100 calcium binding protein A11 (S100A11). (A, B) A2780 and UWB1.289 cells were cultured in medium containing 30 μM and 10 μM olaparib for a duration of 24 h, respectively, and then transferred to fresh medium without olaparib. Protein was extracted and phosphorylation of H2A histone family member X at serine 139 (γH2AX) levels measured at various time points after drug withdrawal (A) and quantification of γH2AX relative protein levels (B). (C, D) Western blot was utilized to quantify S100A11 levels after import S100A11 overexpression plasmid (C) and quantification of S100A11 protein levels (D). (E) A2780 and UWB1.289 cells were cultured in medium containing 30 and10 μM olaparib for a duration of 24 h. Immunofluorescence analysis was employed to detect γH2AX foci at various time intervals following drug withdrawal. The proportion of γH2AX-positive cells is quantified in Figs. S5A and B. (F) Following the discontinuation of olaparib, the recovery rate of γH2AX foci in Fig. 7E was evaluated. (G) Cell Counting Kit-8 (CCK-8) method was utilized to determine the half maximal inhibitory concentration (IC_50_) values of the two ovarian cell types following 72 h of olaparib treatment. ^∗^*P* < 0.05 and ^∗∗^*P* < 0.01. HBLV: HanBio lentivirus; GAPDH: glyceraldehyde-3-phosphate dehydrogenase.

Prior research has indicated that S100A11 is significant in the repair of DNA damage. To further elucidate the functional role of FBXW11 in olaparib-induced DNA damage, A2780 and UWB1.289 cells from both the FBXW11-overexpressing group and the control group were exposed to olaparib for 24 h. After the withdrawal of olaparib, protein samples were collected at different time intervals (0, 6, 12, and 24 h), and Western blotting was performed to quantify γH2AX levels. The results revealed that γH2AX levels were markedly increased in the FBXW11-overexpressing group. Furthermore, the recovery of γH2AX levels was significantly delayed in the FBXW11-overexpressing group, suggesting less efficient DNA damage repair (Figs. 7A and B). To investigate the potential association between the FBXW11-mediated reduction in DNA damage repair efficiency and S100A11, plasmids overexpressing S100A11 were introduced into the FBXW11-overexpressing A2780 and UWB1.289 cells, and transfection efficiency was detected by Western blotting (Figs. 7C and D). We then detected γH2AX foci by immunofluorescence in A2780 and UWB1.289 cells subjected to olaparib treatment for 24 h. In the FBXW11-overexpressing cells, S100A11 overexpression reduced the accumulation of γH2AX foci (Figs. 7E, S5A, and S5B). Next, DNA repair kinetic assays were performed at 0, 6, 12, and 24 h post-olaparib therapy cessation. The FBXW11-overexpressing group showed significantly delayed γH2AX lesion repair; however, after the S100A11 expression plasmid was added, this delay was partially reversed (Fig. 7F). To further determine whether FBXW11 affects ovarian cancer cell sensitivity to olaparib through S100A11, we introduced plasmids overexpressing S100A11 into HBLV group and HBLV FBXW11group (Figs. S5C and D). A CCK-8 assay revealed that the introduction of the S100A11 overexpression plasmid reduced the FBXW11-enhanced susceptibility of ovarian cancer cells to olaparib (Fig. 7G). These results suggest that FBXW11 enhances the degradation of S100A11, thereby reducing the efficiency of DNA damage repair and ultimately promoting the cytotoxic effect of olaparib on ovarian cancer cells.

### 3.7 FBXW11 promotes olaparib sensitivity *in vivo*

**Fig. 8:**
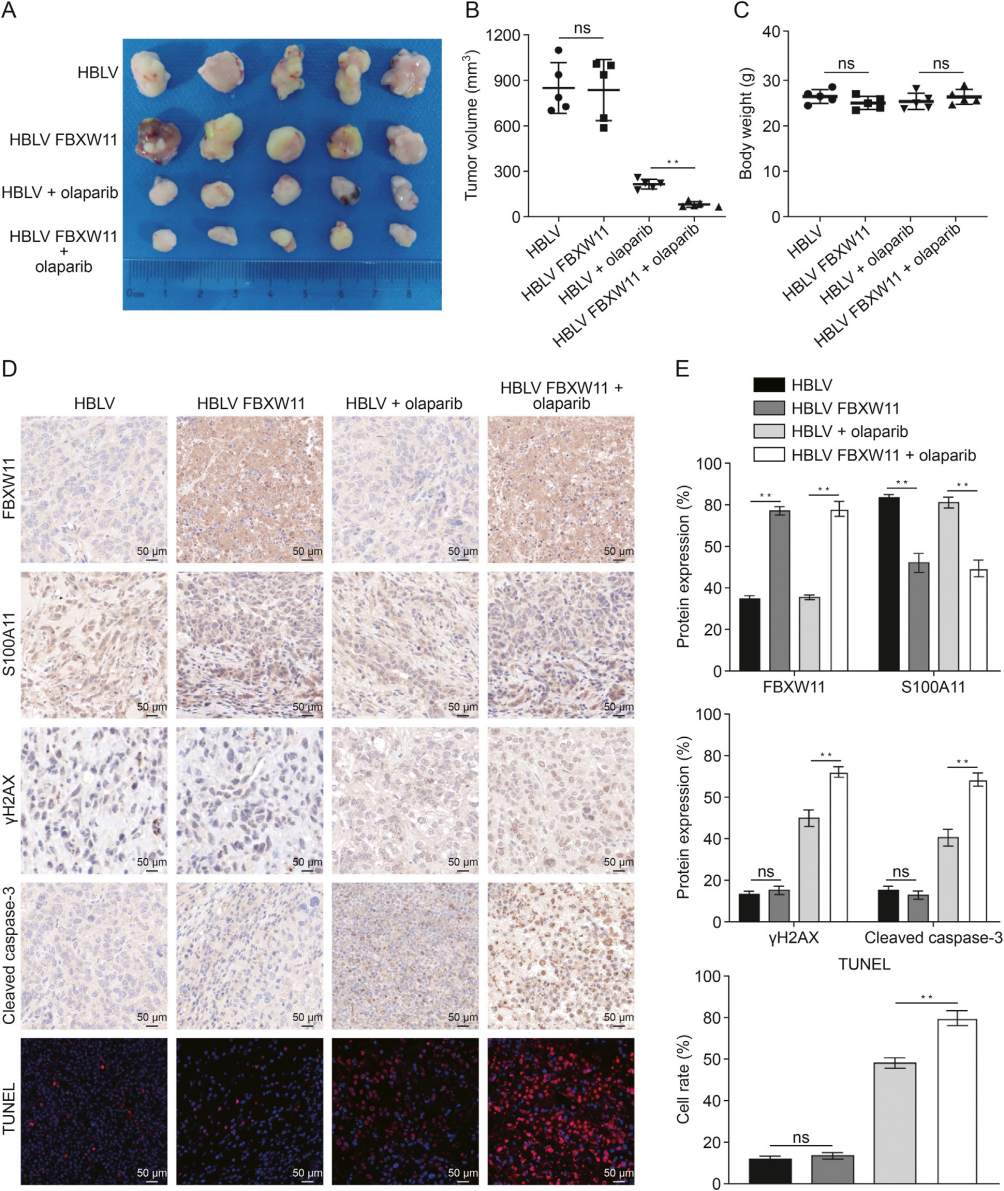
F-box and tryptophan-aspartic (WD) repeat domain containing 11 (FBXW11) increased the killing effect of olaparib on ovarian tumors in mice. (A–C) Each mouse received a subcutaneous injection of 5 × 10^6^ SKOV3 cells transfected with HanBio lentivirus (HBLV) or HBLV FBXW11 in the left axilla. When the tumor volumes attained roughly 50 mm^3^, the mice were distributed at random into four groups (HBLV, HBLV FBXW11, HBLV + olaparib, and HBLV FBXW11 + olaparib) and administered an intraperitoneal dose of olaparib (50 mg/kg) or phosphate-buffered saline (PBS) daily. Mice were euthanized 20 days post-injection, and tumor volume were assessed (A) and quantitative analyses of tumor volume (B) and body weight of the nude mice (C). (D, E) Typical images of immunohistochemistry (IHC) staining for FBXW11, S100 calcium binding protein A11 (S100A11), phosphorylation of H2A histone family member X at serine 139 (γH2AX), cleaved caspase-3, and terminal deoxynucleotidyl transferase (TdT) dUTP nick-end labeling (TUNEL) were observed in tumor tissues (D) and quantifying protein expression levels and TUNEL-positive cell rates (E). ^∗∗^*P* < 0.01. ns: not significant.

We subcutaneously inoculated BALB/c nude mice with SKOV3 cells from the HBLV group and the HBLV FBXW11 group to evaluate the *in vivo* impact of FBXW11. When the tumor volume reached 50 mm^3^, the mice were randomly divided into olaparib treatment group and PBS treatment group, and received continuous treatment for a duration of 20 days. Compared with the HBLV group, the HBLV FBXW11 group presented significantly smaller tumors, indicating the ability of FBXW11 to increase the toxic effect of olaparib on ovarian cancer cells *in vivo* (Figs. 8A and B). Mouse body weight was not affected by olaparib treatment or FBXW11 overexpression (Fig. 8C). γH2AX and cleaved caspase-3 levels were significantly higher in the HBLV FBXW11 group than in the HBLV group. Moreover, the HBLV FBXW11 group had a higher proportion of positive TUNEL staining. These findings indicate that the overexpression of FBXW11 enhances olaparib-induced apoptosis and DNA damage (Figs. 8D and E). Therefore, our research results show that FBXW11 can promote PARPi sensitivity *in vivo*.

## 4 Discussion

Accumulating evidence suggests that FBXW11 has differential expression patterns across various tumor types and plays a variety of roles in human cancer by targeting different substrates. However, there are no relevant reports on FBXW11 expression in ovarian cancer or its function in ovarian cancer treatment. Within our research, we present the first evidence of FBXW11 expression in HGSOC patients and its association with patient prognosis. For the first time, we demonstrated that FBXW11 functions by modulating the S100A11-mediated DNA damage repair pathway to enhance ovarian cancer cells' susceptibility to PARPis. Our results show that FBXW11 exhibits promising potential as a novel biomarker for predicting both the prognosis of ovarian cancer patients and their sensitivity to PARPis.

In recent years, PARPi, such as olaparib, have received approval for treating different types of cancer including ovarian, breast, prostate, and pancreatic cancers. However, similar to the case for many other anticancer therapies, patients often eventually develop resistance despite a robust initial response to PARPi. Therefore, understanding the mechanisms of PARPi resistance and identifying reliable biomarkers to predict PARPi sensitivity are essential prerequisites for overcoming PARPi resistance. Owing to alterations in the microenvironment and specific target substrates in different types of cancer, FBXW11 exerts dual effects, both promoting and inhibiting cancer progression. This study reveals, for the first time, that FBXW11 contributes to increased sensitivity to PARP inhibitors in BRCA mutant and wild-type ovarian cancer cells. The overexpression of FBXW11 markedly enhanced the sensitivity of ovarian cancer cells to PARPi, while the knockdown of FBXW11 facilitated resistance to PARP inhibitors. Experiments in the xenograft models further validated that FBXW11 overexpression promoted the therapeutic effect of PARPi in ovarian cancer *in vivo*. Precise upregulation of FBXW11 has the potential to enhance the clinical response of ovarian cancer to PARPi.

Currently, the most extensively investigated mechanisms of PARPi resistance encompass restoring homologous recombination (HR) capacity, augmenting drug efflux, diminishing PARP1 trapping, and stabilizing replication forks [29]. The restoration of HR capability constitutes the primary determinant of clinical resistance to platinum-based chemotherapy and PARP inhibitors [30]. In response to DNA damage, PARP1 is rapidly recruited to sites of breaks, subsequently facilitating the recruitment of BRCA1 and BRCA2 [31]. BRCA1 facilitates DNA double-strand break end resection and collaborates with BRCA2 and PALB2 to increase RAD51 recruitment to resected single-stranded DNA, thereby promoting HR repair [32,33]. Therefore, targeting pivotal molecules involved in HR repair is promising for enhancing PARPi sensitivity by impeding DNA damage repair. S100A11 accelerates reparation of breaks by increasing RAD51 activity and regulating RAD51 persistence at double-strand break repair sites. In non-small cell lung cancer, S100A11 knockdown significantly augments tumor sensitivity to cisplatin, oxaliplatin, and 5-fluorouracil. Similarly, S100A11 knockdown enhances the susceptibility of gastric cancer to 5-fluorouracil and cisplatin. In ovarian cancer, S100A11 promotes tumor invasion and metastasis, and high S100A11 expression levels are associated with poor PFS and OS. However, studies on the correlation between S100A11 and the sensitivity of ovarian cancer to PARPi are lacking. Our study in ovarian cancer demonstrated that FBXW11 increased the ubiquitination and degradation of S100A11, resulting in less efficient DNA damage repair and consequently heightened sensitivity to PARPi. We hypothesized that the underlying mechanism might involve decreased S100A11-mediated double-stranded DNA repair due to the increased degradation of S100A11. Relevant investigations will be conducted in subsequent studies to validate this hypothesis.

## 5 Conclusion

In our research, we confirmed that FBXW11 expression is markedly decreased in HGSOC tissues, and that diminished FBXW11 expression serves as a prognostic indication of adverse outcomes and medication resistance in HGSOC patients. Additionally, FBXW11 facilitates the degradation of S100A11 via ubiquitination, thereby increasing the sensitivity of ovarian cancer cells to PARPi. The combination of FBXW11 overexpression or S100A11 inhibition with PARPi might have great potential in the medical therapy of ovarian cancer.

However, there are some limitations to our study. Given the *in vitro* culture of ovarian cancer cells, which lacks the tumor microenvironment crucial for their survival *in vivo*, it is important to acknowledge that research findings on their functions may exhibit certain deviations from the physiological conditions observed *in vivo*. Although we have used BALB/c nude mice to verify that FBXW11 can also promote the susceptibility of ovarian cancer to olaparib *in vivo*, due to many differences in physiological structure between mice and humans, more clinical trials may be needed for further verification.

## CRediT authorship contribution statement

**Ligang Chen:** Writing – review & editing, Writing – original draft, Visualization, Validation, Supervision, Software, Resources, Project administration, Formal analysis, Data curation. **Mingyi Wang:** Writing – original draft, Visualization, Validation, Supervision, Software, Resources, Project administration. **Yunge Gao:** Supervision, Software, Project administration, Methodology. **Yanhong Lv:** Resources, Project administration, Methodology, Investigation. **Lianghao Zhai:** Visualization, Validation, Supervision, Software. **Jian Dong:** Resources, Methodology, Formal analysis. **Yan Chen:** Software. **Xia Li:** Project administration, Conceptualization. **Xin Guo:** Resources, Project administration. **Biliang Chen:** Project administration, Methodology, Investigation, Funding acquisition, Formal analysis, Data curation, Conceptualization. **Yi Ru:** Resources, Project administration, Methodology, Investigation, Conceptualization. **Xiaohui Lv:** Methodology, Investigation, Funding acquisition, Formal analysis, Conceptualization.

## Declaration of competing interest

The authors declare that there are no conflicts of interest.
