# Supplementary material for: E3 ubiquitin ligase FBXW11-mediated downregulation of S100A11 promotes sensitivity to PARP inhibitor in ovarian cancer
Source: J Pharm Anal. 2025 Feb 27;15(7):101246. doi: 10.1016/j.jpha.2025.101246 (PMC12311512; doi:10.1016/j.jpha.2025.101246)
Supplement: Multimedia component 1 [file mmc1.docx]

Supplementary figures


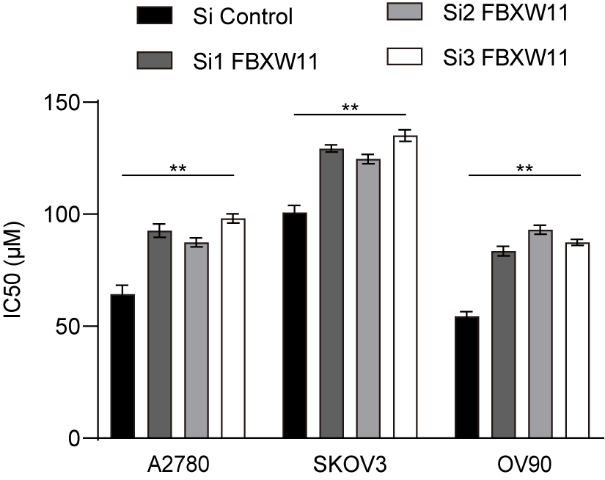


Fig.S1 Supplementary Figure for Fig.1. Statistical analysis of the Half maximal inhibitory concentration (IC_50_) values in Fig. 1F. ^**^*P* < 0.01. si: small interfering; FBXW11: F-box and tryptophan-aspartic (WD) repeat domain containing 11.


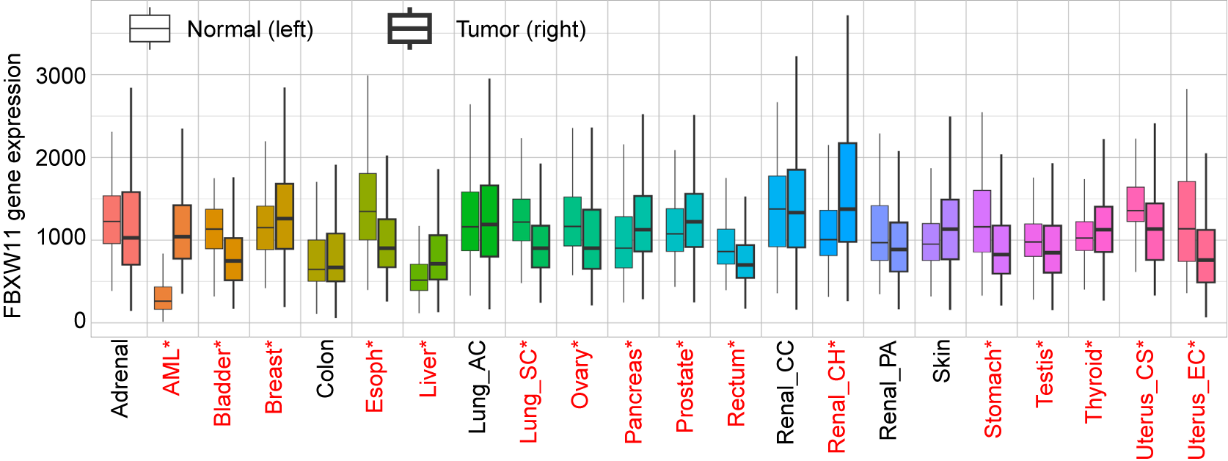


Fig.S2 Supplementary Figure for Fig.2. F-box and tryptophan-aspartic (WD) repeat domain containing 11 (FBXW11) is expressed at low levels in a variety of human tissue cancers, including ovarian cancer. The data were obtained from The Cancer Genome Atlas (TCGA) database. ^*^*P* < 0.05.


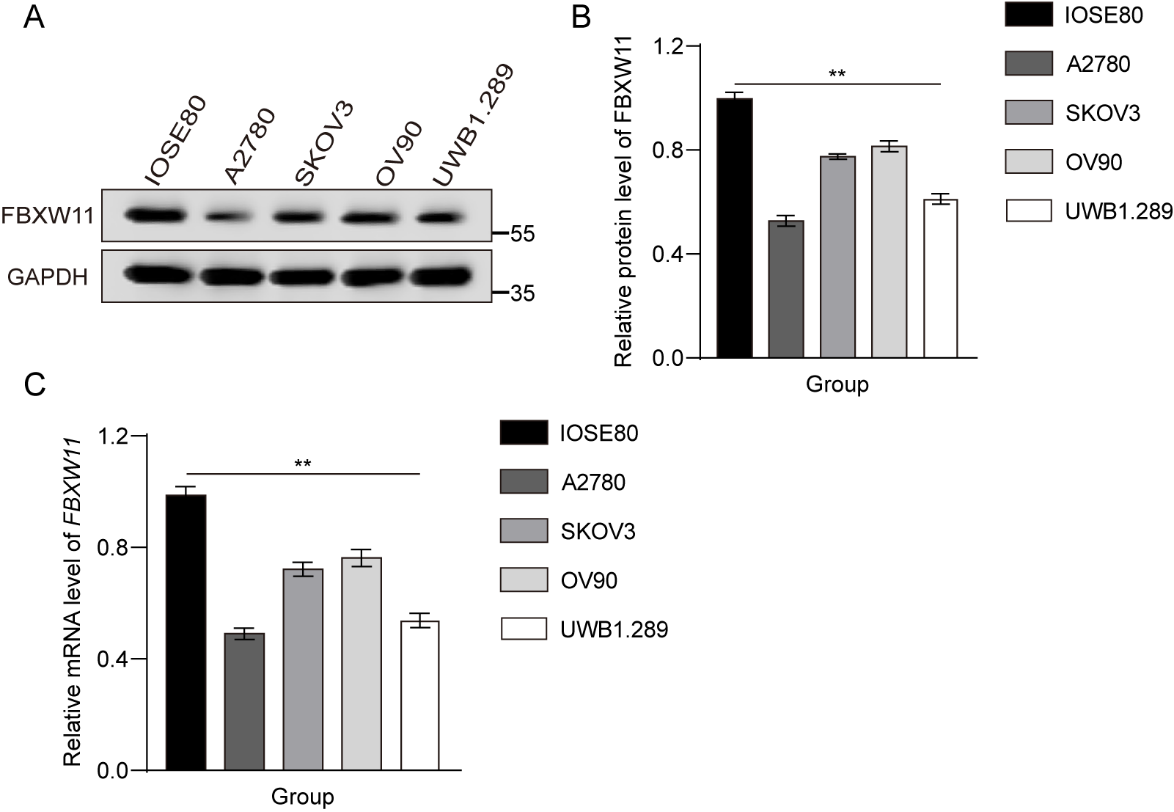


Fig.S3 Supplementary Figure for Fig.3. (A, B) The protein expression levels of F-box and tryptophan-aspartic (WD) repeat domain containing 11 (FBXW11) in these four ovarian cancer cell lines and the normal ovarian cell line IOSE80 were assessed using Western blot analysis (A) and quantification of FBXW11 protein levels (B). (C) Quantitative real-time reverse transcription polymerase chain reaction (qRT-PCR) was used to detect the mRNA levels of *FBXW11* in these four ovarian cancer cell lines and one normal ovarian cell line. ^**^*P* < 0.01. GAPDH: glyceraldehyde-3-phosphate dehydrogenase.


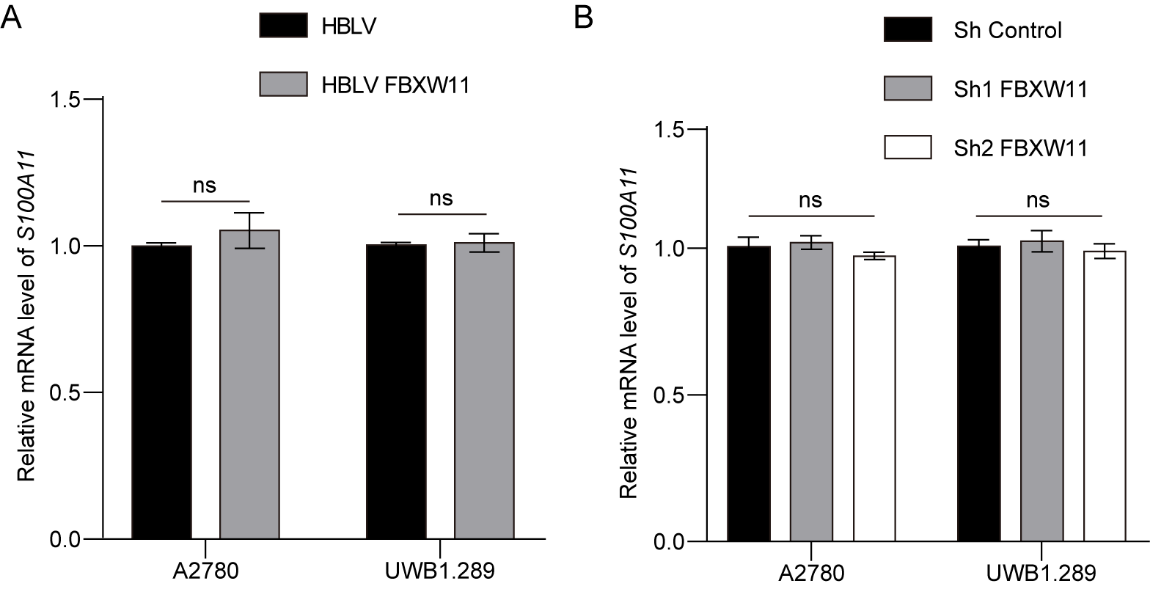


Fig.S4 Supplementary Figure for Fig.6. (A, B) Quantitative real-time reverse transcription polymerase chain reaction (qRT-PCR) was used to detect the mRNA level of S100 calcium binding protein A11 (*S100A11*) in F-box and tryptophan-aspartic (WD) repeat domain containing 11 (FBXW11) overexpression group (A) and FBXW11 knockdown group (B). ns: not significant. sh: short hairpin.


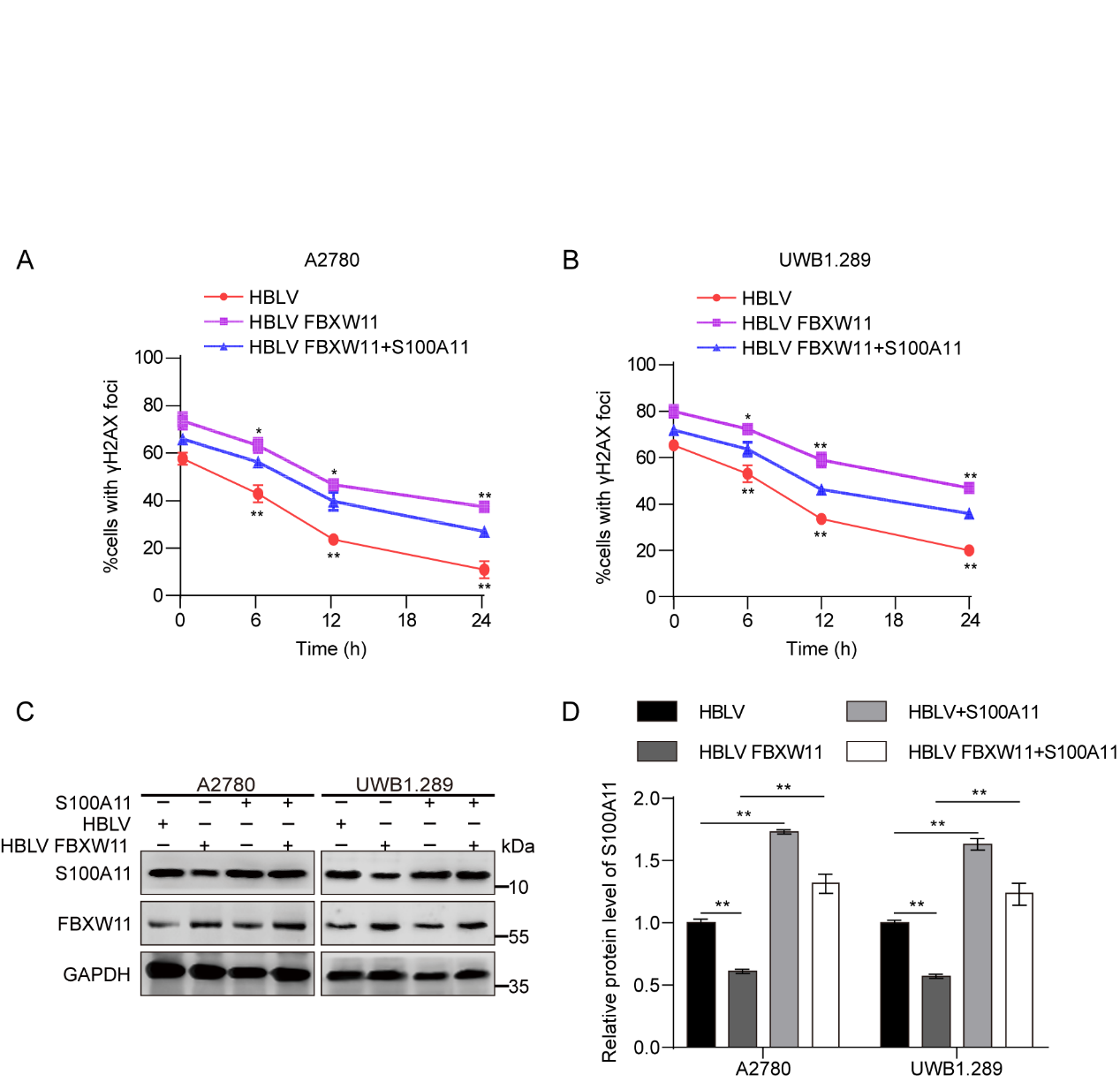


Fig.S5 Supplementary Figure for Fig.7. (A, B) Quantification of the proportion of phosphorylation of H2A histone family member X at serine 139 (γH2AX)-positive cells in Fig. 7E**:** On the left is the quantification of the A2780 group (A) and on the right is the quantification of UWB1.289 group (B). (C, D) The S100 calcium binding protein A11 (S100A11) plasmids were transfected into the HBLV group and HBLV F-box and tryptophan-aspartic (WD) repeat domain containing 11 (FBXW11) group of A2780 cells and UWB1.289 cells, followed by Western blot analysis to detect the levels of S100A11 protein after 48 h (C) and quantification of protein levels (D). ^*^*P* < 0.05 and ^**^*P* < 0.01. GAPDH: glyceraldehyde-3-phosphate dehydrogenase.
