# Supplementary material for: E3 ubiquitin ligase FBXW11-mediated downregulation of S100A11 promotes sensitivity to PARP inhibitor in ovarian cancer
Source: J Pharm Anal. 2025 Feb 27;15(7):101246. doi: 10.1016/j.jpha.2025.101246 (PMC12311512; doi:10.1016/j.jpha.2025.101246)
Supplement: Multimedia component 2 [file mmc2.pdf]

非标记定量泛素化蛋白质组学

（泛素化 4D Label-free）实验方法说明

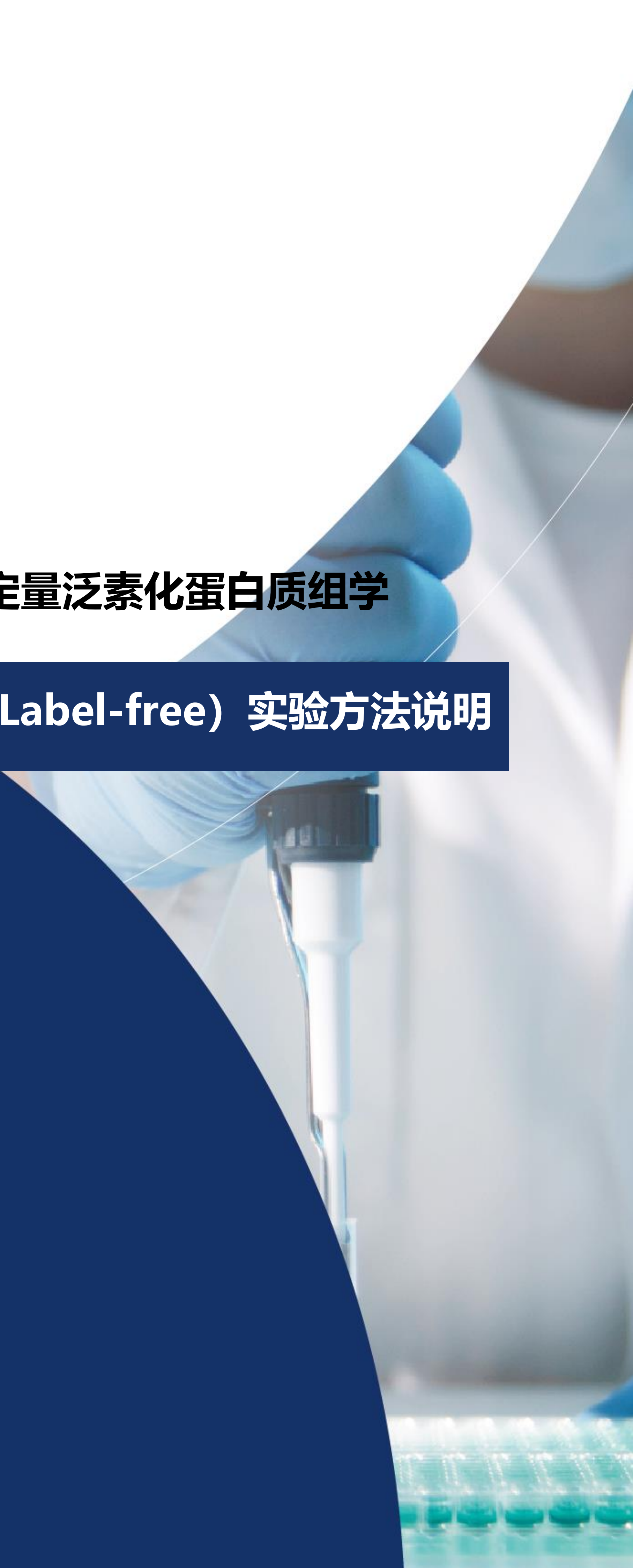

目 录

1. 4D Label-free 原理 3

2. 泛素化肽段富集 4

3. 项目流程 4

4. 实验方法 5

4.1 实验仪器和分析软件 5

4.2 试剂和耗材 5

4.3 蛋白质抽提 7

4.4 SDS-PAGE 电泳 8

4.5 FASP 酶解 9

4.6 泛素化肽段富集 9

4.7 质谱分析 10

4.7.1 NanoElute 色谱 10

4.7.2 质谱鉴定 10

4.8 数据分析 11

4.8.1 质谱文件处理 11

4.8.2 数据库选择 12

4.8.3 蛋白质定性和定量分析参数 12

5. 参考文献 13

1. 4D Label-free 原理

非标记定量蛋白质组学（Label-free）技术是一种不依赖于同位素标记的蛋白质定量技术。传统的质谱分离鉴定包括三个维度，即离子强度（intensity），质荷比（m/z）和保留时间（retention time）。因此传统的 labelfree 蛋白质组学实验中，在有限的扫描速率下，仪器只能选择离子强度高的肽段进行检测，从而在实验中产生了一定的随机性并影响整体的蛋白质鉴定通量。而在添加了离子淌度维度之后，肽段在电场的作用下依据分子的形状和截面进行分离，从而将 m/z 维度上无法分离的肽段进行进一步分离，降低图谱的复杂度，提高鉴定通量与鉴定速度。这使得 timsTOF Pro 能够拥有更好的灵敏度，更加适用于高通量蛋白质组学研究。

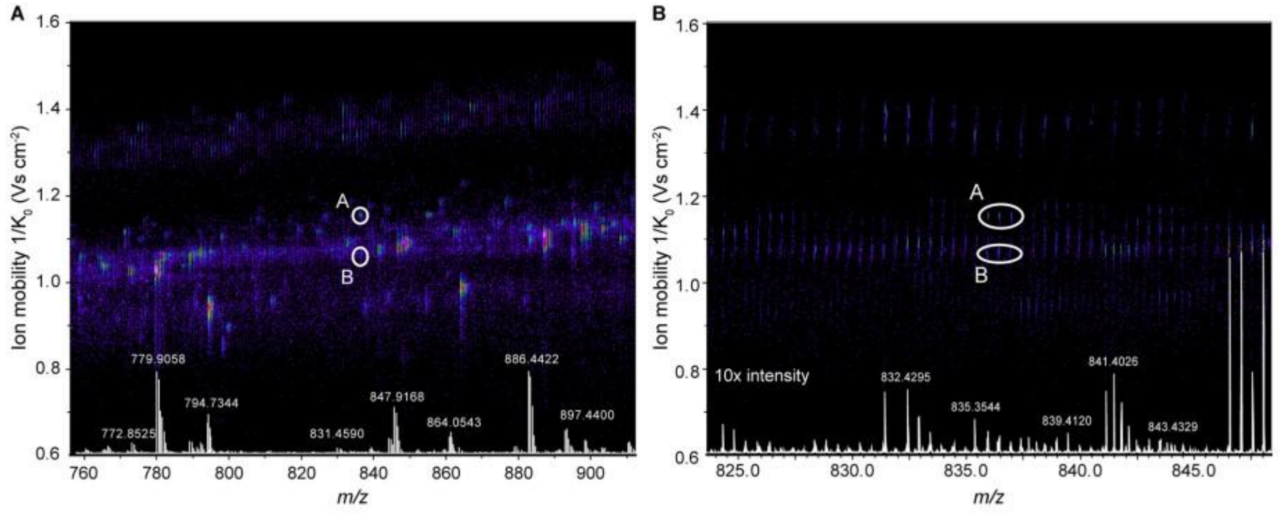

timesTOP Pro 中肽段离子在离子淌度维度下进行分离

同时，labelfree 分析软件 MaxQuant 软件对 4D 蛋白质定量技术进行了全面升级（针对 MBR-ddaPASEF），利用了离子淌度维度 CCS 数值，有效地提高强化 MBR 的功能，从而保证了传统 shotgun 蛋白质组学数据的平行性，可靠性与特异性，减少定量的 missing value，提升鉴定深度。

2. 泛素化肽段富集

泛素化是指泛素（一类低分子量的蛋白质）分子在一系列特殊的酶作用下，将细胞内的蛋白质分类，从中选出靶蛋白分子，并对靶蛋白进行特异性修饰的过程。这些特殊的酶包括泛素激活酶，结合酶、连结酶和降解酶等。泛素化在蛋白质的定位、代谢、功能、调节和降解中都起着十分重要的作用。同时，它也参与了细胞周期、增殖、凋亡、分化、转移、基因表达、转录调节、信号传递、损伤修复、炎症免疫等几乎一切生命活动的调控。泛素化与肿瘤、心血管等疾病的发病密切相关。因此，作为近年来生物化学研究的一个重大成果，它已然成为研究、开发新药物的新靶点。

带有泛素化修饰的蛋白质丰度相对较低，因此需先对泛素化肽段进行富集。最常见且高效的泛素化肽段富集方法是用对泛素化赖氨酸（K-GG）具有高亲和力的基序抗体进行富集。本项目蛋白质经酶解后利用对泛素化赖氨酸（K-GG）具有高亲和力的基序抗体对泛素化肽段进行富集，然后通过 LC-MS/MS 对泛素化修饰位点进行分析，从而实现大规模泛素化蛋白质的定性定量分析。

3. 项目流程

泛素化 4D Label-free 技术的优势在于不需要对样本进行复杂标记或者处理，即可实现对多组样本进行蛋白质定量及显著性差异分析。本项目流程分为预实验和正式实验两部分：预实验包括蛋白质提取、蛋白质定量、SDS-PAGE、蛋白质酶解步骤；正式实验是在预实验的基础上进行的，对预实验中质控合格的样本采用高分辨质谱仪进行正式实验，获得质谱原始数据。

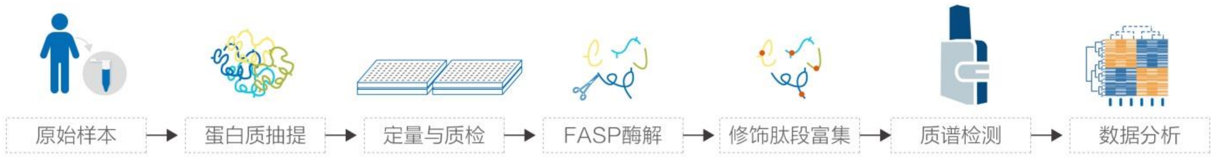

泛素化 4D Label-free 定量蛋白质组学实验流程图

4. 实验方法

4.1 实验仪器和分析软件

实验仪器：	
➢ NanoElute 色谱系统（Bruker, Bremen, Germany)<br>➢ Agilent 1260 infinity II HPLC 系统<br>➢ 低温高速离心机（Eppendorf 5430R)<br>➢ 电泳仪（BIO-RAD)<br>➢ 超声破碎仪（宁波新芝 JY96-IIN)<br>➢ Votex 振荡器（GENIE Votex-2)<br>➢ Nano Drop（Thermo Fisher scientific ND2000）	➢ timsTOF Pro 质谱仪（Bruker, Bremen, Germany)<br>➢ Multiskcan FC 酶标仪（Thermo Fisher Scientific)<br>➢ 真空离心浓缩仪（太仓华美 LNG-T98)<br>➢ MP Fastprep-24 匀浆仪（MP Fastprep-24 5G)<br>➢ 恒温培养箱（上海精宏 GNP-9080）<br>➢ 电子天平（OHAUS AX324Z)<br>➢ 精巧型恒温混匀仪（大龙 HCM-100 pro）
**分析软件：**	
➢ Perseus 1.3 (Max Planck Institute of Biochemistry in Martinsried, Germany)<br>➢ MaxQuant 1.6.17.0	➢ R version 3.3.1

4.2 试剂和耗材

➢ Urea (BIO-RAD，161-0731)	➢ SDS-PAGE 蛋白上样缓冲液（碧云天，P0015F**)**

➢ SDS (生工，SB0485-500g)<br>➢ Tris (生工，T0826-500g)<br>➢ 碘乙酰胺（IAA , Sigma，I1149-5G)<br>➢ C18 Empore™ 固相萃取圆盘（Sigma，66883-U）<br>➢ BCA 定量试剂盒（碧云天，P0012）<br>➢ NH_4HCO_3 (Sigma，A6141-25G)<br>➢ 甲酸（Thermo Fisher Scientific, A117）<br>➢ 乙腈 (Merck，1000304008)<br>➢ C18 Cartridge (Waters，WAT023590)<br>➢ Acetyl-Lysine Motif [Ac-K] Kit (Cell Signaling Technology ,13416S)<br>➢ 1M TEAB（Thermo SE252676/90114）<br>➢ Thermo Scientific High SelectTM Top14 Abundant Protein Depletion Mini Spin Columns (Thermo)	➢ Lysing Matrix A(MP, 6910-100-99219)<br>➢ 二硫苏糖醇 (DTT, Sigma， 43819-5G)<br>➢ $HCOONH_4$ (Sigma, 17843)<br>➢ NH_3•H_2O(Sigma, 17837)<br>➢ HCl（国药，10011018）<br>➢ BSA（生工， A0332）<br>➢ Trypsin (Promega, V5117)<br>➢ 三氟乙酸（TFA , Sigma, T6508）<br>➢ 30kD 超滤离心管 (Sartorius, VN01H22)<br>➢ 0.22 µm 超滤离心管（Corning Spin-X, 8160）<br>➢ Multiple Affinity Removal LC Column – Human 14 / Mouse 3 (Agilent)
C18 分析柱：IonOpticks, Australia，25cm X 75µm, 1.6µm C18 beads	
SDT 裂解液：4%SDS，100mM Tris-HCl，pH 7.6	
UA buffer：8M Urea，150mM Tris-HCl，pH 8.5	
NanoElute 流动相 A：0.1%FA	

NanoElute 流动相 B：0.1% FA, 100% ACN

4.3 蛋白质抽提

具体选择以下哪种裂解方法参见项目方案或者预实验结果处。

i. TCA/丙酮沉淀+SDT 裂解法[1]：

适用样品类型：植物组织（根、茎、叶等）、动物坚硬组织（皮肤、软骨、毛发等）、真菌

取适量样本在液氮中用研钵磨碎成细粉状，加入 5 倍体积的 TCA/丙酮（1:9），涡旋混匀，置于-20℃沉淀 4h 以上。4℃ 6000g 离心 40min，弃上清。加入预冷丙酮，洗涤三次。通风橱中干燥沉淀。称取 20-30mg 干燥后的粉末，加入 30 倍体积（m/v）的 SDT 裂解液，Votex 重悬沉淀，沸水浴 5min。超声破碎，沸水浴 15min。14000g 离心 15min，取上清采用 0.22 μm 滤膜过滤，收集滤液。采用 BCA 法进行蛋白质定量。分装样品，-80℃保存。

对应英文描述：

The samples were frozen in liquid nitrogen and ground with a pestle and mortar. 5 times volume of TCA/acetone (1:9) was added to the powder and mixed by vortex. The mixture was placed at -20°C for 4h, and centrifuged at 6000g for 40 min at 4°C. The supernatant was discarded. The pre-cooling acetone was added and washed for three times. The precipitation was air dried. 30 times volume of SDT buffer (4%SDS，100mM Tris-HCl，pH 7.6) was added to 20-30 mg powder, mixed and boiled for 5 min. The lysate was sonicated and then boiled for 15 min. After centrifuged at 14000g for 15 min, the supernatant was filtered with 0.22 μm filters. The filtrate was quantified with the BCA Protein Assay Kit (P0012, Beyotime). The sample was stored at -80 °C.

ii. 匀浆+SDT 裂解法[2]：

适用样品类型：动物柔软组织（脑、肝、肌肉等）、软体动物、微生物菌体等

取组织或微生物菌体沉淀加入适量 SDT 裂解液，转移至 Lysing Matrix A 管中，应用 MP 匀浆仪进行匀浆破碎（24×2， 6.0M/S，30s，两次）。超声后，沸水浴 10min。14000g 离心 15min，取上清采用 0.22 μm 离心管过滤，收集滤液。采用 BCA 法进行蛋白质定量。分装样品，-80℃保存。

对应英文描述：

SDT buffer was added to the sample, and transferred to 2 ml tubes with amount quartz sand. The lysate was homogenized by MP Fastprep-24 Automated Homogenizer (6.0M/S, 30s, twice). The homogenate was sonicated and then boiled for 10 min. After centrifuged at 14000g for 15 min, the supernatant was filtered with 0.22 μm filters. The filtrate was quantified with the BCA Protein Assay Kit (P0012, Beyotime). The sample was stored at -80 °C.

iii. SDT 裂解法[3]：

适用样品类型：细胞、蛋白质沉淀粉末、体液、浓缩后的发酵液或者细胞分泌上清等。

取样品加入适量 SDT 裂解液，超声（溶液类样品此操作可跳过），沸水浴 15min。14000g 离心 15min，取上清。采用 BCA 法进行蛋白质定量。分装样品，-80℃保存。

对应英文描述：

SDT buffer (4%SDS， 100mM Tris-HCl， pH 7.6) was added to the sample. The lysate was sonicated (this step can be skipped for protein solution) and then boiled for 15 min. After centrifuged at 14000g for 15 min, the supernatant was quantified with the BCA Protein Assay Kit (P0012, Beyotime). The sample was stored at -80 °C.

4.4 SDS-PAGE 电泳

各样品取蛋白质 20 μg 分别加入 6X 上样缓冲液，沸水浴 5min，进行 12% SDS-PAGE 电泳（恒压 250V，40min），考马斯亮蓝染色。

对应英文描述：

20 μg of proteins for each sample were mixed with 6X loading buffer respectively and boiled for 5 min. The proteins were separated on 12% SDS-PAGE gel. Protein bands were visualized by Coomassie Blue R-250 staining.

4.5 FASP 酶解

各样品取 5 mg 蛋白质溶液，分别加入 DTT 至终浓度为 100mM，沸水浴 5min，冷却至室温。加入 200 μL UA buffer 混匀，转入 30kD 超滤离心管中，离心 12500g 15min，弃滤液（重复该步骤一次）。加入 100 μL IAA buffer（100mM IAA in UA），600rpm 振荡 1min，室温避光反应 30min，离心 12500g 15min。加入 100μL UA buffer 离心 12500g 15min，重复该步骤两次。加入 100μL 50mM NH_4HCO_3 溶液，离心 12500g 15min，重复该步骤两次。换新收集管，加入 40μL Trypsin buffer（4μg Trypsin in 40μL 50mM NH_4HCO_3 溶液），600rpm 振荡 1min，37°C 放置 16-18h。离心 12500g 15min；再加入 40μL 50mM NH_4HCO_3 溶液，离心 12500g 15min，收集滤液。采用 C_{18} Cartridge 对肽段进行脱盐，肽段冻干后加入 40μL 0.1%甲酸溶液复溶，肽段定量（OD280）。

对应英文描述：

5 mg of proteins for each sample were reduced with 100 mM DTT for 5 min at 100 ℃. Then the detergent, DTT and other low-molecular-weight components were removed using UA buffer (8 M Urea, 150 mM Tris-HCl pH 8.5) by repeated ultrafiltration (Sartorius, 30 kD). Then 100 μl iodoacetamide (100 mM IAA in UA buffer) was added to block reduced cysteine residues and the samples were incubated for 30 min in darkness. The filters were washed with 100 μl UA buffer three times and then 100 μl 50 mM NH_4HCO_3 buffer twice.Finally, the protein suspensions were digested with 4 μg trypsin (Promega) in 40 μl 50 mM NH_4HCO_3 buffer overnight at 37 ℃, and the resulting peptides were collected as a filtrate. The peptide segment was desalted by C_{18} column. The peptide content was estimated by UV light spectral density at 280 nm using an extinctions coefficient of 1.1 of 0.1% (g/l) solution that was calculated on the basis of the frequency of tryptophan and tyrosine in vertebrate proteins.

4.6 泛素化肽段富集

参照 PTMScan（R）HS [K-epsilon-GG] Kit (Cell Signaling Technology, 59322S)的说明手册进行富集。

对应英文描述：

The peptides mixture was subjected to PTMScan（R）HS [K-epsilon-GG] Kit (Cell Signaling Technology, 59322S) for Kac enrichment.

4.7 质谱分析

4.7.1 NanoElute 色谱

样品采用纳升流速 NanoElute 系统进行分离，该系统和装有 CaptiveSpray 离子源的质谱仪 timsTOF Pro 连用。缓冲液 A 液为 0.1%甲酸水溶液，B 液为 0.1%甲酸乙腈水溶液（乙腈为 100%）。色谱柱以 100%的 A 液平衡，样品由自动进样器上样到分析柱（IonOpticks, Australia, 25cm X 75μm, C18 填料 1.6μm）分离，流速为 300 nL/min。

对应英文描述：

Samples were analyzed on a nanoElute (Bruker, Bremen, Germany) coupled to a timsTOF Pro (Bruker, Bremen, Germany) equipped with a CaptiveSpray source. Peptides were separated on a 25cm X 75μm analytical column, 1.6μm C18 beads with a packed emitter tip (IonOpticks, Australia). The column temperature was maintained at 50°C using an integrated column oven (Sonation GmbH, Germany). The column was equilibrated using 4 column volumes before loading sample in 100% buffer A (99.9% MilliQ water, 0.1% FA) (Both steps performed at 800bar). Samples were separated at 300nl/min using a linear gradient.

4.7.2 质谱鉴定

样品经色谱分离后用 timsTOF Pro (Bruker, Bremen, Germany)质谱仪的 PASEF 模式进行质谱分析。分析时长为 120min，检测方式为正离子，母离子扫描范围 100-1700 m/z，离子淌度 1/K0 的范围是 0.75-1.4 V·s/cm2，离子累积或释放时间 100ms，离子利用率为 100%，毛细管电压 1500V，干燥气体速度 3L/min，

干燥温度 180 ℃。PASEF 的设置为：10 个 MS/MS scans（总的循环时间是 1.16s），电荷范围 0-5，动态排除时间 0.5min，离子目标强度 10000，离子强度阈值 2500，CID 碎裂能量 20-59 eV。

对应英文描述：

The timsTOF Pro (Bruker, Bremen, Germany) was operated in PASEF mode. Mass Range 100 to 1700m/z, 1/K0 Start 0.75 V·s/cm2 End 1.4 V·s/cm2, Ramp time 100ms, Lock Duty Cycle to 100%, Capillary Voltage 1500V, Dry Gas 3 l/min, Dry Temp 180 ℃, PASEF settings: 10 MS/MS scans (total cycle time 1.16sec), charge range 0-5, active exclusion for 0.5 min, Scheduling Target intensity 10000, Intensity threshold 2500, CID collision energy 20-59eV.

4.8 数据分析

4.8.1 质谱文件处理

Maxquant 是领先的蛋白质组学定性定量算法，近年来已经逐渐成为蛋白质组学领域内的标准解决方案之一。在蛋白质组学研究中，高分辨率质谱仪采集原始数据的方式可以分为数据依赖性采集（Data-dependent acquisition, DDA）和数据非依赖性采集（Data-independent acquisition, DIA）两种。基于 Label-free 技术的蛋白质定量分析一般采用 DDA 方式进行原始数据的采集，也就是普通意义上的 shotgun 方式。在 DDA 模式的采集方式中，质谱仪先采集一次 MS1 数据，然后采集若干次 MS2 数据。在本实验中，采用的是基于 MS1 数据积分的非标记定量方法。由于 MS1 的数据密度非常高，因此在数据积分计算之前，还需要对原始数据进行 peptide feature 的智能识别工作。Maxquant 软件的工作流程就是按照先识别 peptide feature，然后进行图形积分计算强度值进行的。

Maxquant 经过多次升级，现在已经高度集成化、智能化。在 Label-free 实验的数据分析阶段，完全实现了智能化一键操作。在完成质谱的原始文件采集后，只需设定好分组情况、数据库和翻译后修饰类型，即可直接进行操作。Maxquant 不仅提供非标记定量的强度数据，还可以自动进行数据库匹配，获得蛋白质

的定性结果。在 Maxquant 的定性匹配中，算法采用 FDR 原理进行数据筛选，获得高度可信的定性结果。此外，定性的序列信息和定量信息可以在软件中自动关联，形成统一的表格文件，方便后续分析。

4.8.2 数据库选择

选择适当的蛋白质序列数据库是对质谱数据进行蛋白质定性分析的基础和关键步骤。通常数据库建立的数据来源主要有以下几种：

1) 综合性蛋白质数据库，如 NCBInr、UniProt 等；

2) 特定物种的蛋白质数据库，如拟南芥（TAIR）、水稻（RAP DB）、家蚕（silkdb）等；

3) 已测序物种由测序结果翻译而成的蛋白质序列数据。

本项目使用数据库为：uniprot_homo_20230312_20423_9606_swiss_prot

（下载链接：http://www.uniprot.org）

4.8.3 蛋白质定性和定量分析参数

本项目采用 MaxQuant 软件（版本号 1.6.17.0）进行数据库搜索。相关参数和说明如下:

Item	Value
➢ **Enzyme**	● Trypsin
➢ **Max Missed Cleavages**	● 2
➢ **Main search**	● 10 ppm
➢ **First search**	● 20 ppm
➢ **MS/MS Tolerance**	● 20 ppm
➢ **Fixed modifications**	● Carbamidomethyl (C)
➢ **Variable modifications**	● Oxidation (M), K(GG)

➢ **Database**	➢ uniprot_homo_20230312_20423_9606_swiss_prot
➢ **Database pattern**	● Target-Reverse
➢ **Include contaminants**	● True
➢ **Peptide FDR**	● ≤0.01
➢ **Protein FDR**	● ≤0.01

对应英文描述：

The MS data were analyzed using MaxQuant software version 1.6.17.0. MS data were searched against the database(**determined by project**). An initial search was set at a precursor mass window of 6 ppm. The search followed an enzymatic cleavage rule of Trypsin/P and allowed maximal two missed cleavage sites and a mass tolerance of 20ppm for fragment ions. Carbamidomethylation of cysteines was defined as fixed modification, while protein N-terminal acetylation and methionine oxidation were defined as variable modifications for database searching. The cutoff of global false discovery rate (FDR) for peptide and protein identification was set to 0.01. Protein abundance was calculated on the basis of the normalized spectral protein intensity (LFQ intensity). Proteins which Fold change>2 or <0.5 and p value (Student′ s t test) <0.05 were considered to be a differentially expressed protein.

5. 参考文献

[1] Thiellement H , Zivy M , Damerval , et al. Plant Proteomics: Methods and Protocols[J]. Methods in Molecular Biology, 355.

[2] Zhu Y , Xu H , Chen H , et al. Proteomic Analysis of Solid Pseudopapillary Tumor of the Pancreas Reveals Dysfunction of the Endoplasmic Reticulum Protein Processing Pathway[J]. Molecular & Cellular Proteomics, 2014. 13(10):2593-603.

[3] WisNiewski J R , Zougman A , Nagaraj N , et al. Universal sample preparation method for proteome analysis[J]. Nature Methods, 2009, 6(5):359-362.

[4] Agilent Multiple Affinity Removal Columns – for Mouse Serum Proteins. Agilent Technologies, Inc. 2005.

[5] Immunodepletion of High-Abundant Proteins from Rat Serum with the Agilent Multiple Affinity Removal System for Mouse. Agilent Technologies, Inc. 2004

未经本实验室批准，部分采用本实验报告内容无效。
Partly using this report will not be admitted unless allowed by Testing Center.
