# Supplementary figures and images for: E3 ubiquitin ligase FBXW11-mediated downregulation of S100A11 promotes sensitivity to PARP inhibitor in ovarian cancer
Source: J Pharm Anal. 2025 Feb 27;15(7):101246. doi: 10.1016/j.jpha.2025.101246 (PMC12311512; doi:10.1016/j.jpha.2025.101246)

Fig.1D

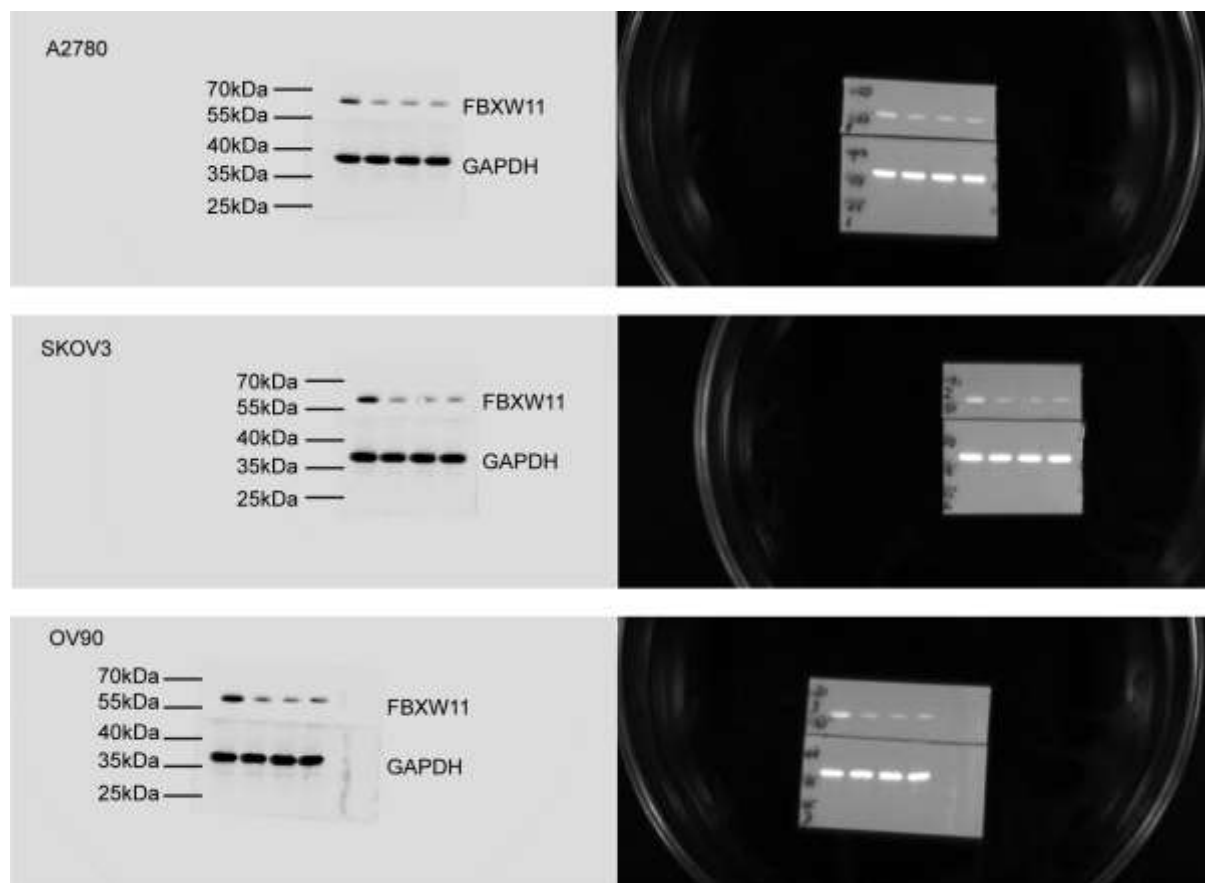

Fig.2B

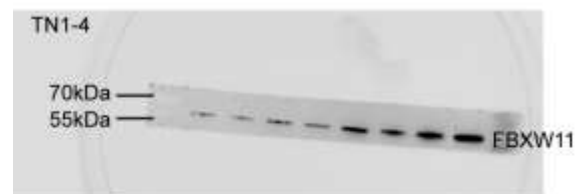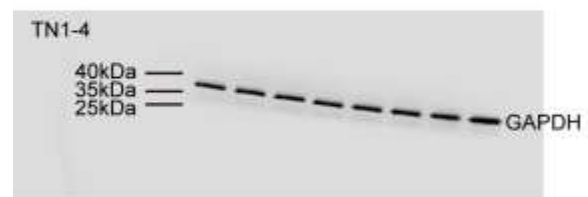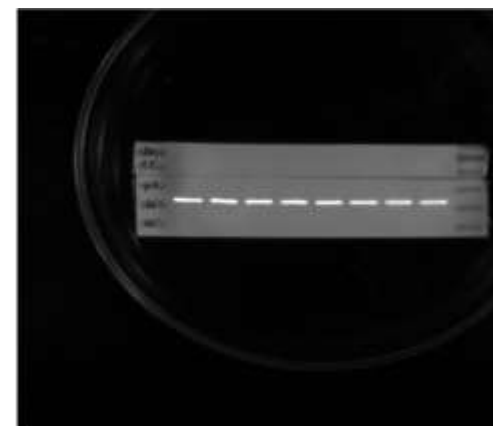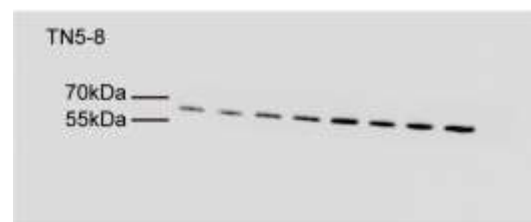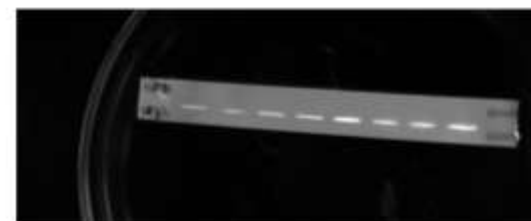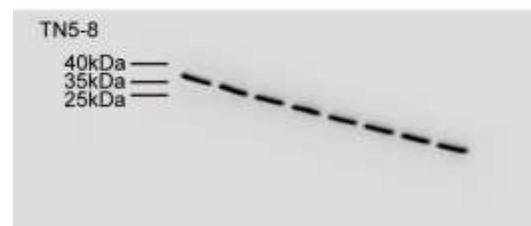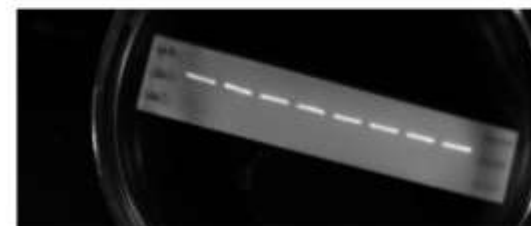

Fig.3A

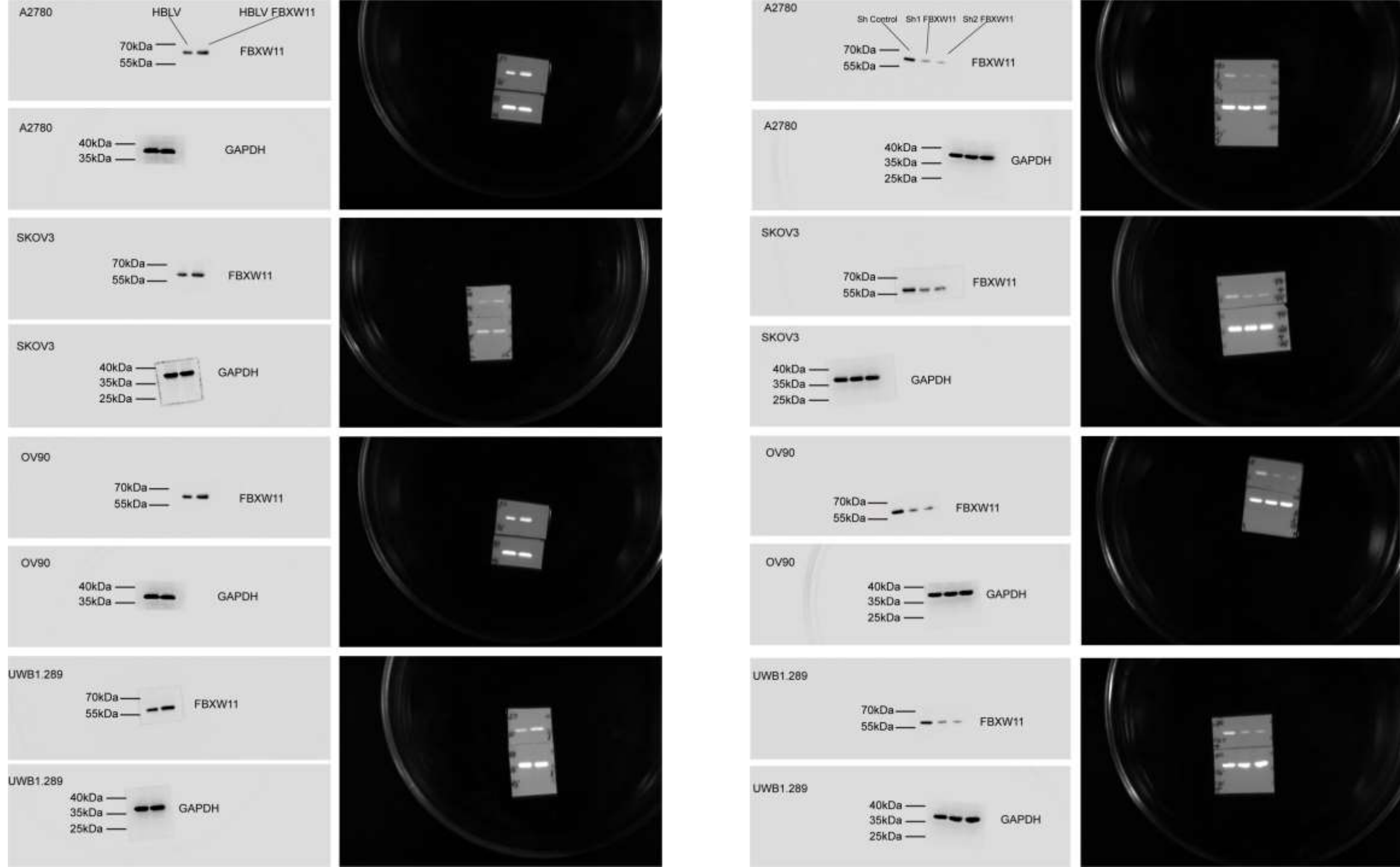

Fig.4E

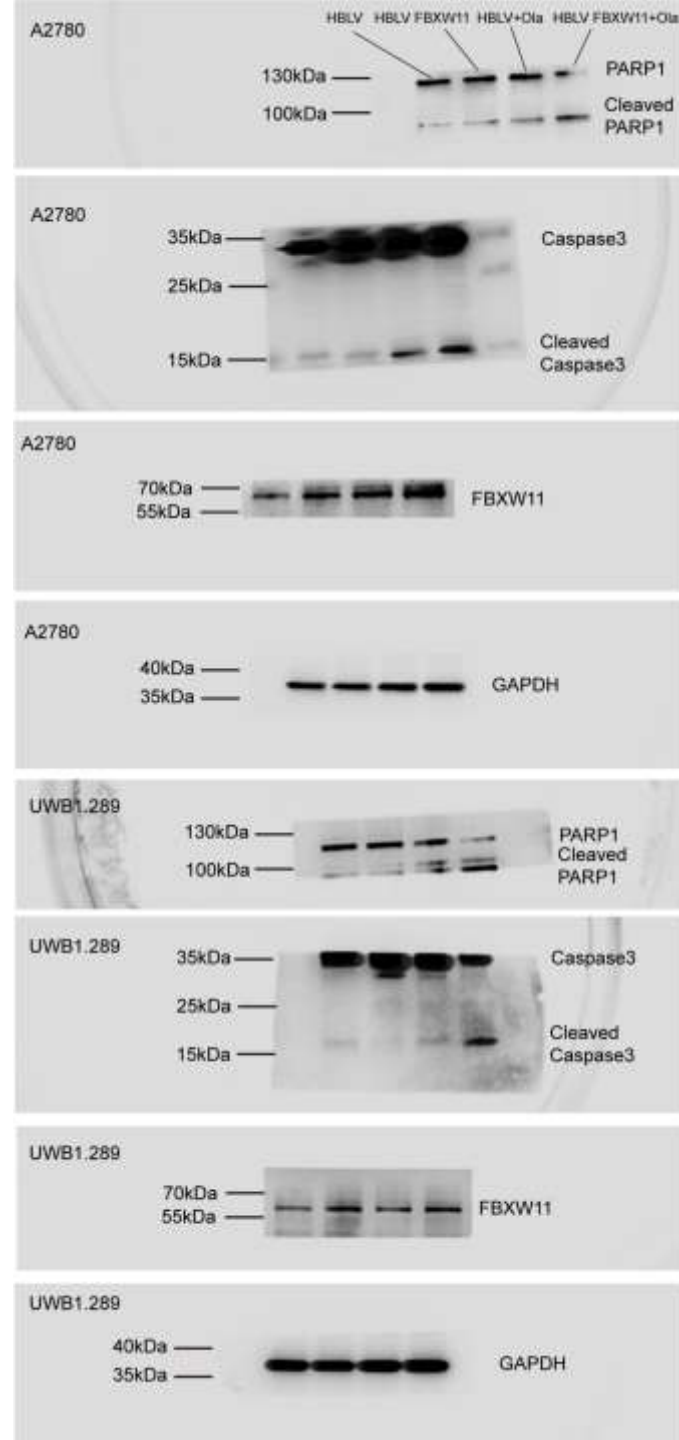

Fig.5B

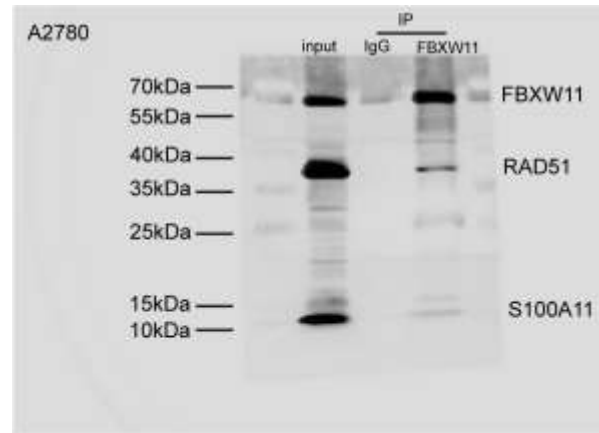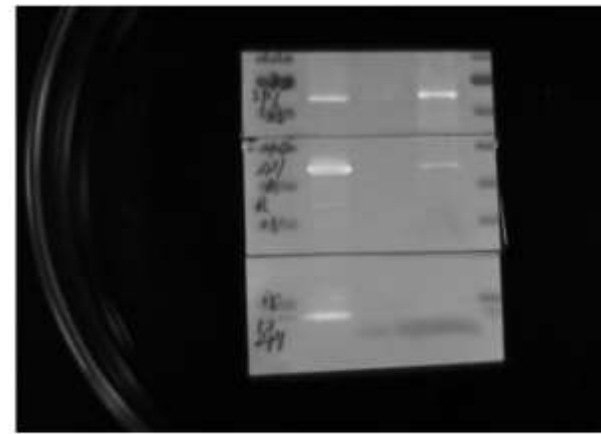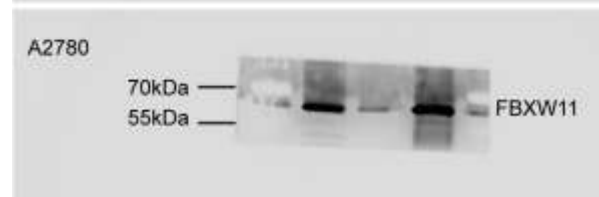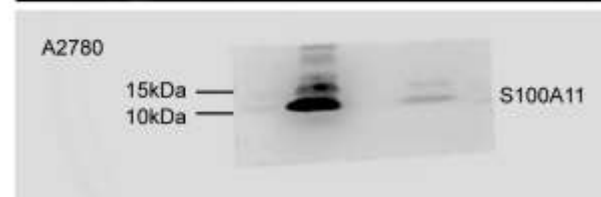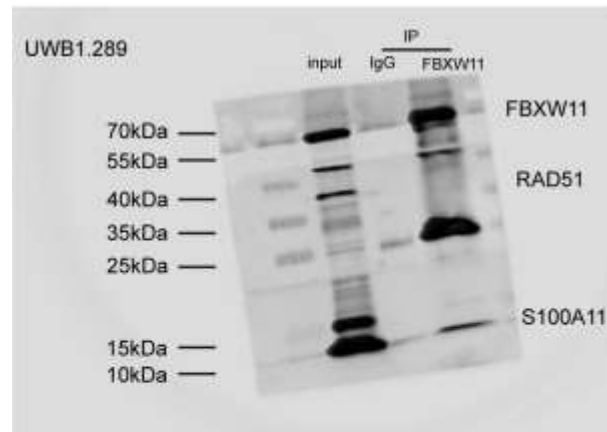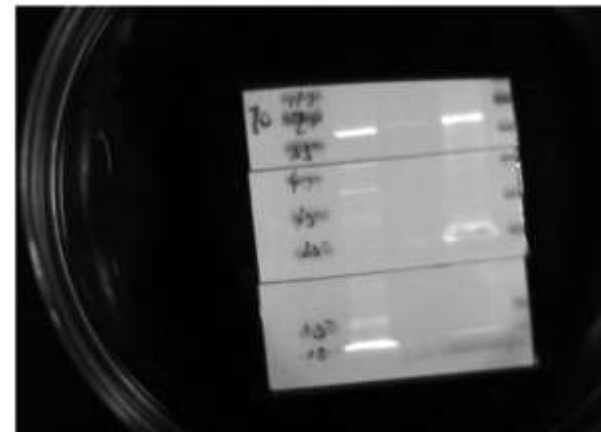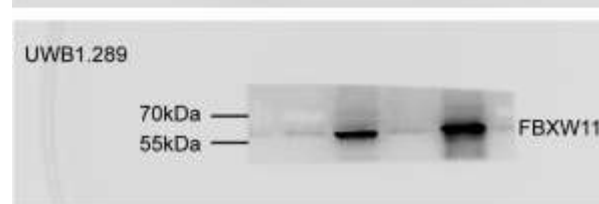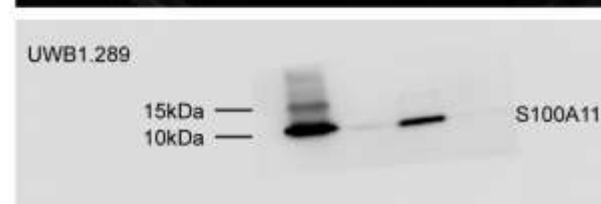

Fig.5D

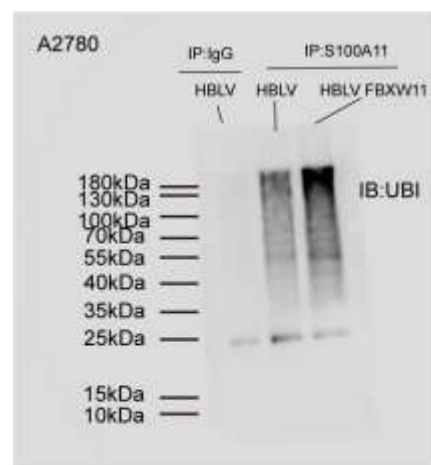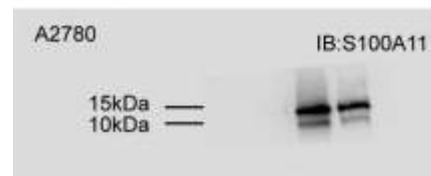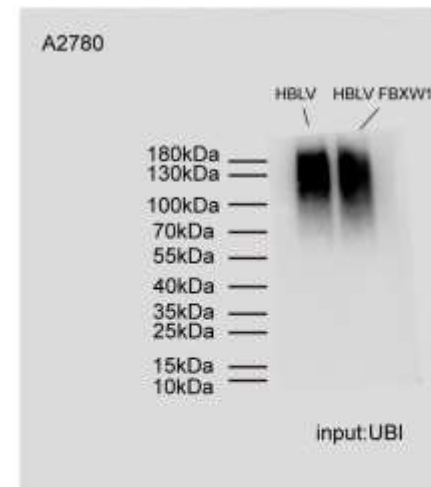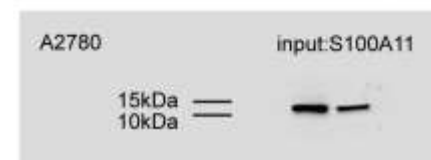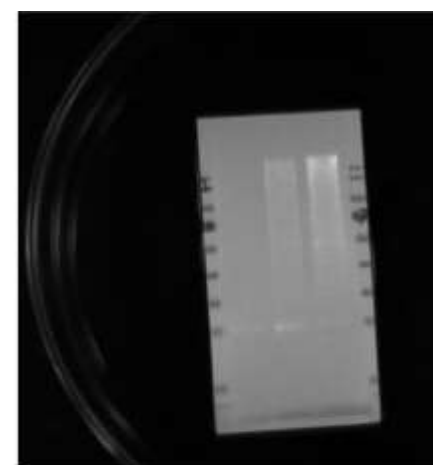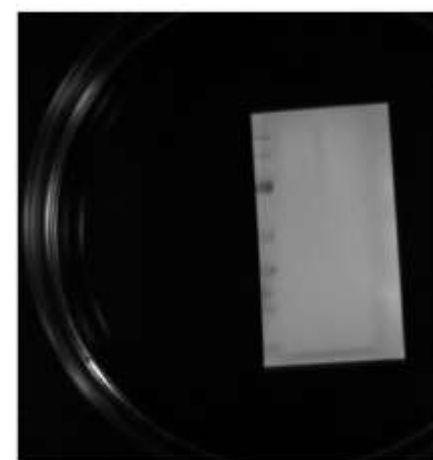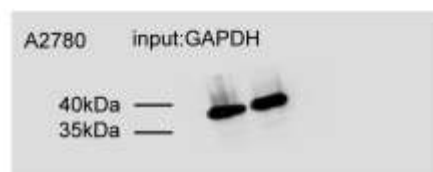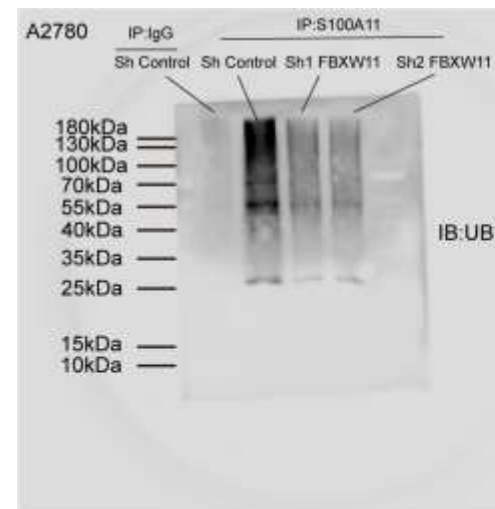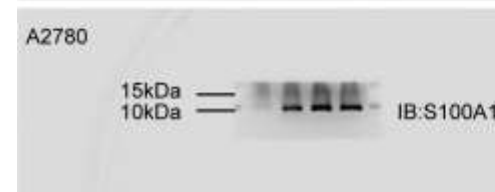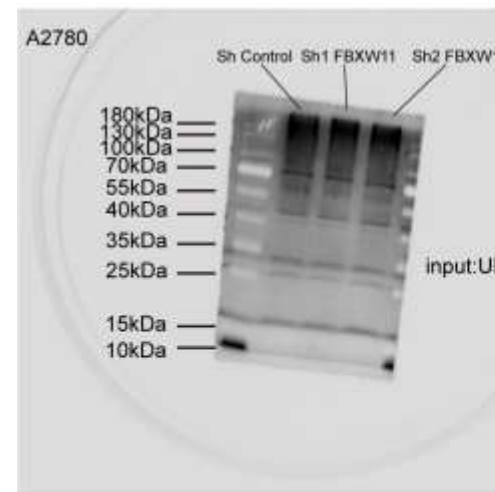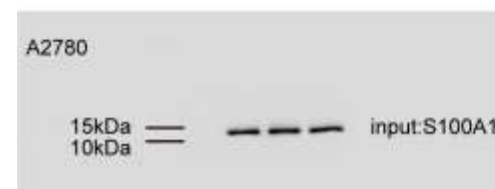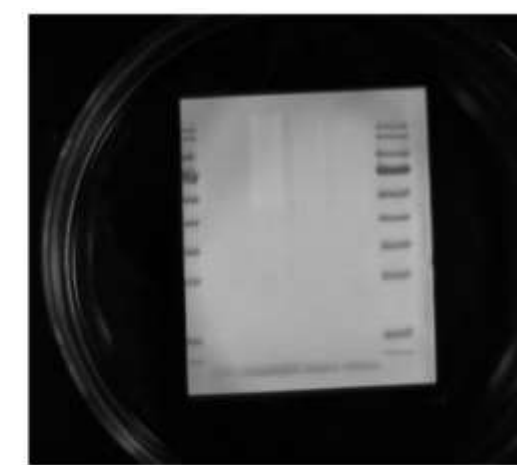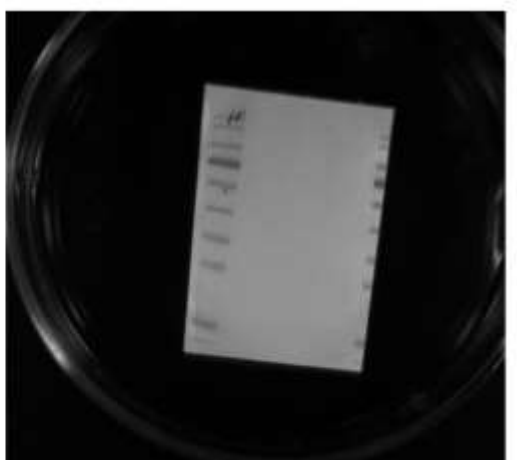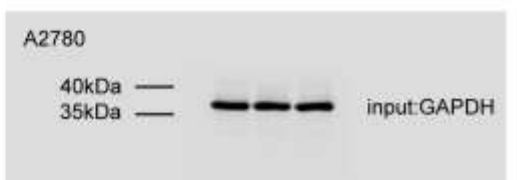

Fig.5D

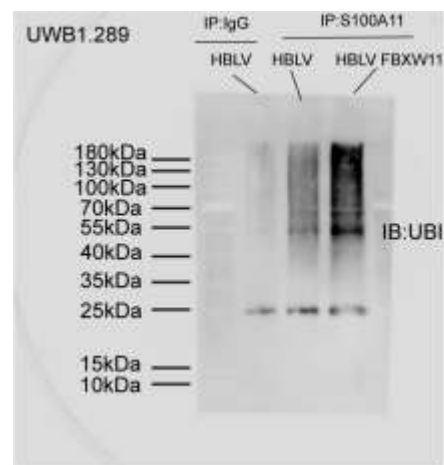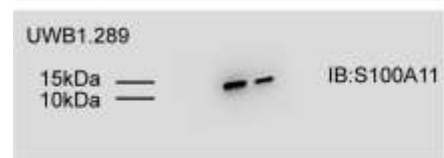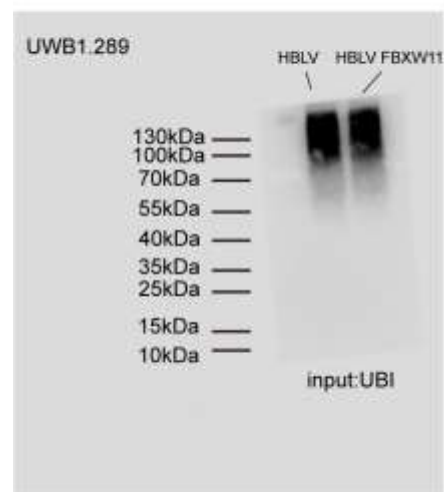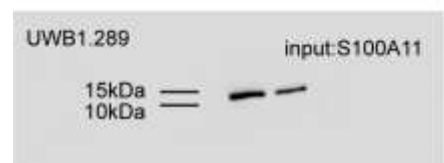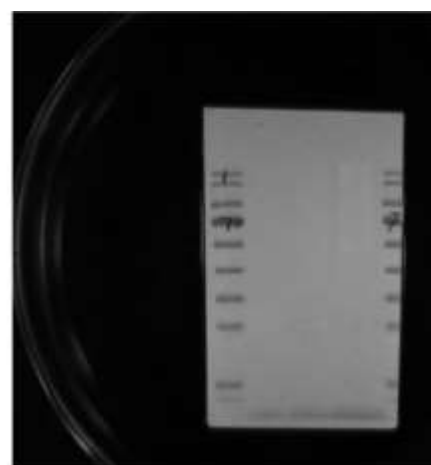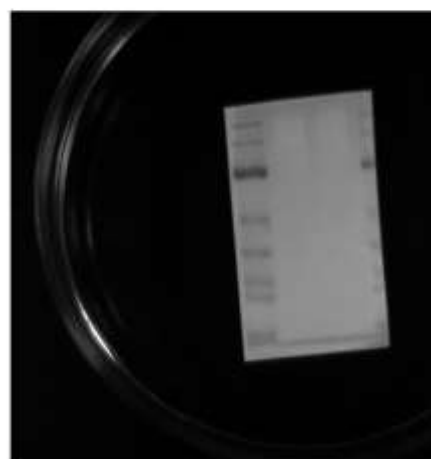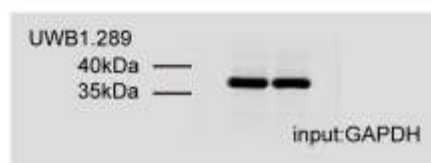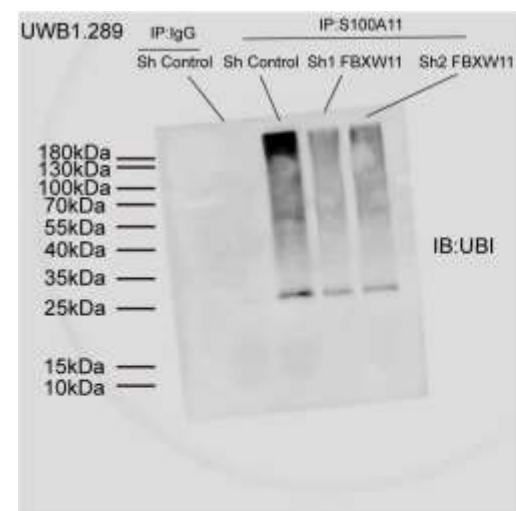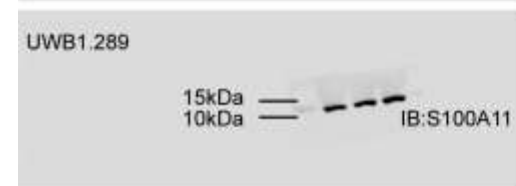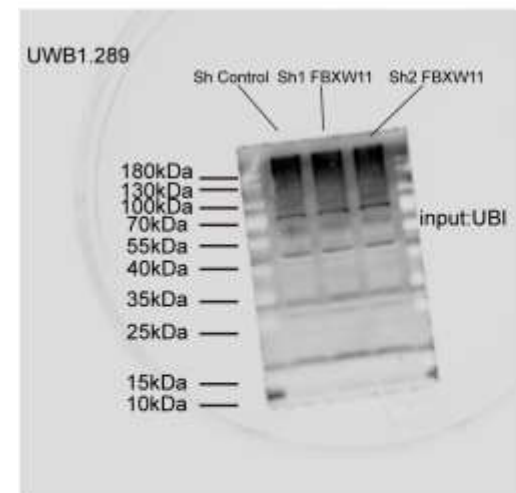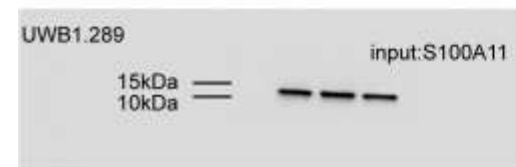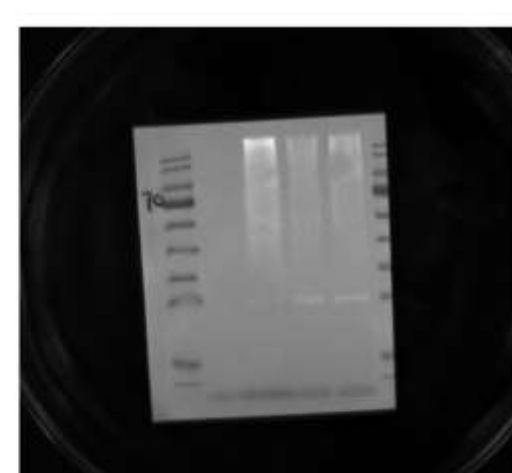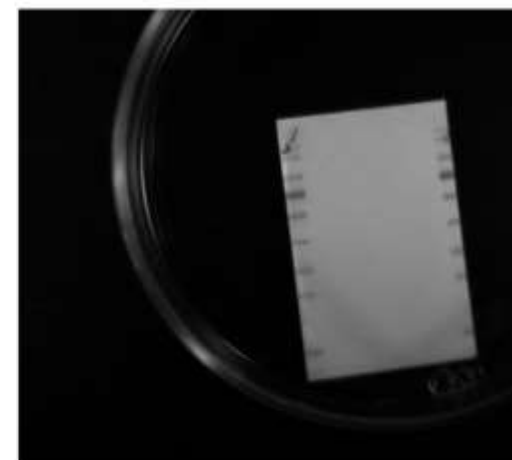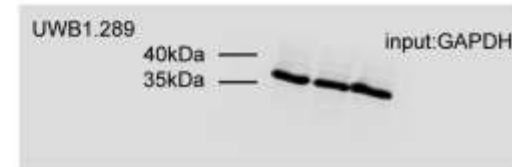

Fig.6A

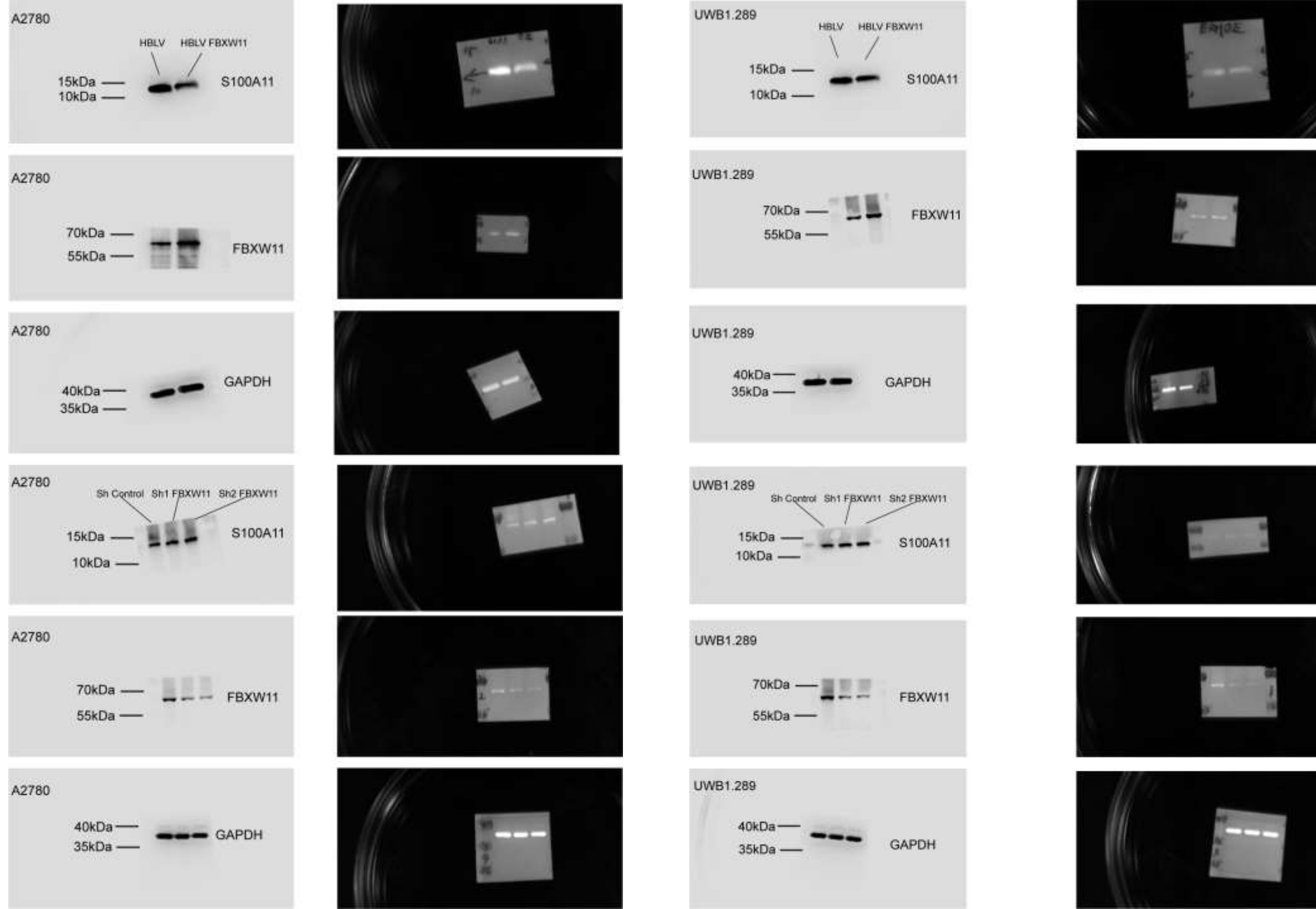

Fig.6D

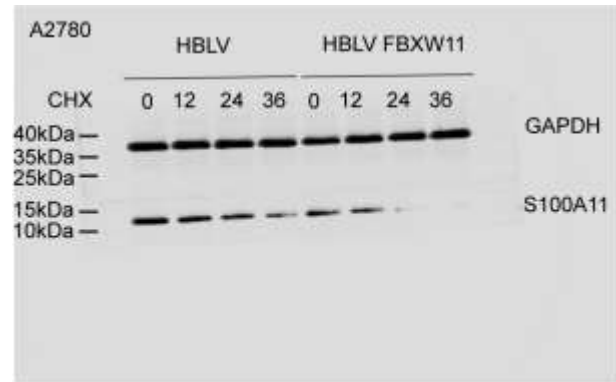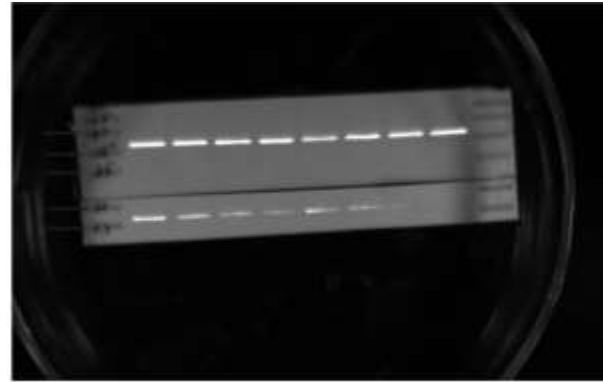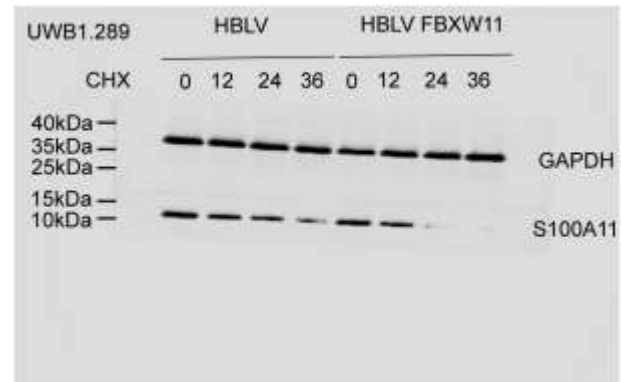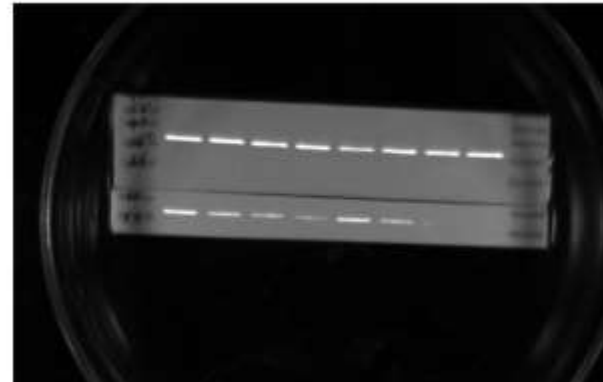

Fig.6F

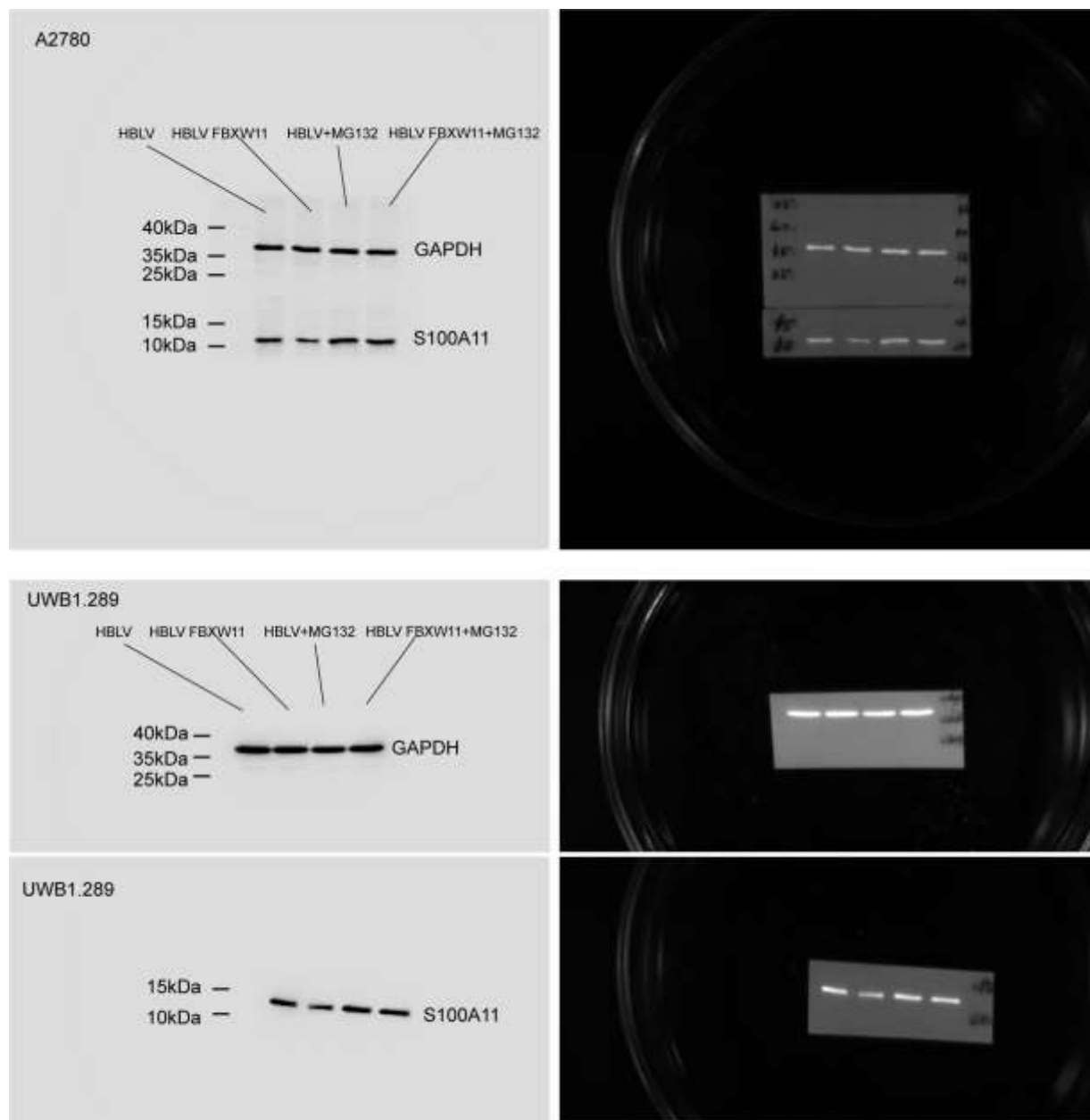

Fig.7A

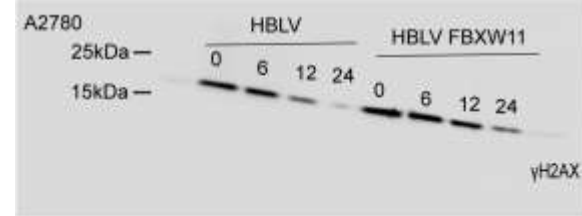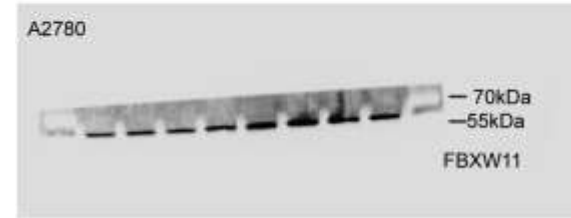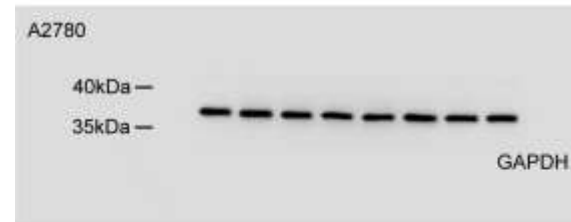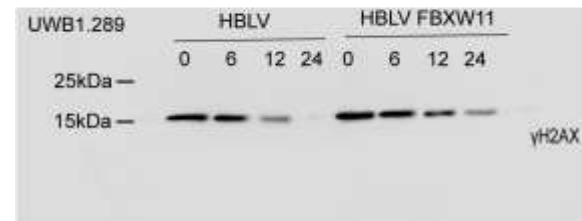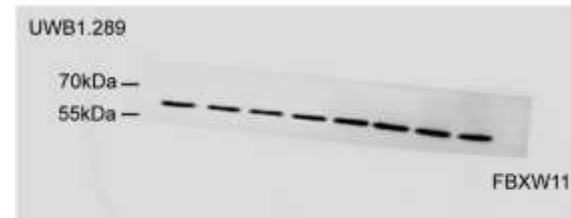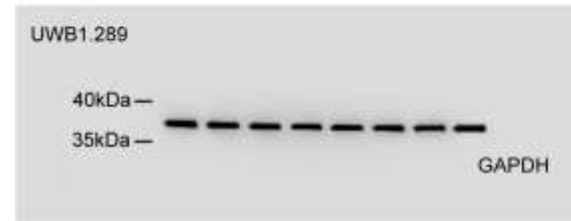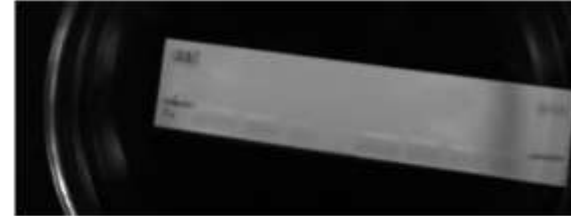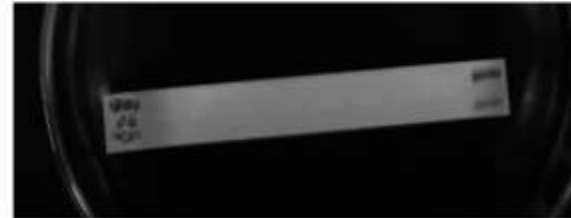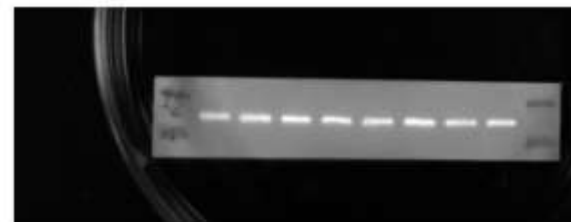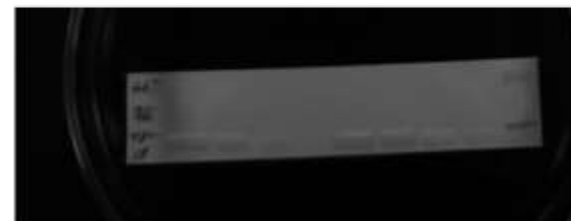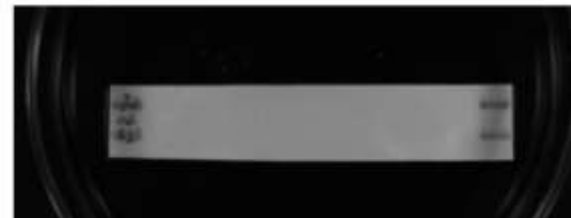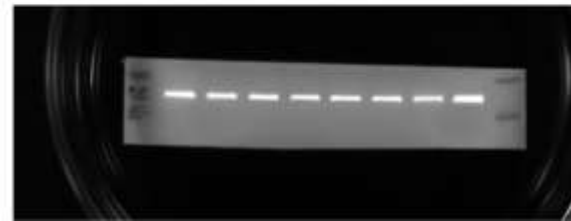

Fig.7C

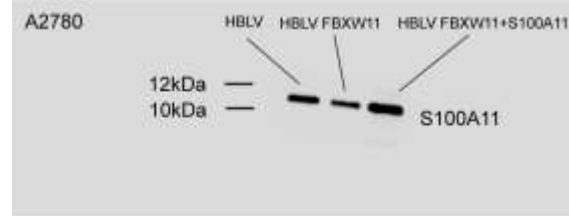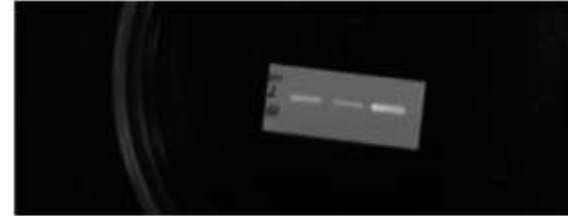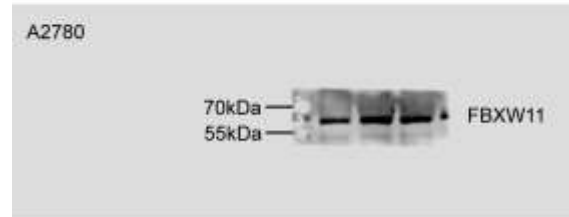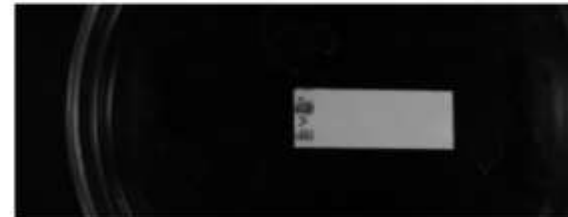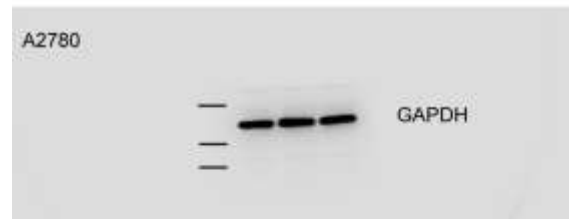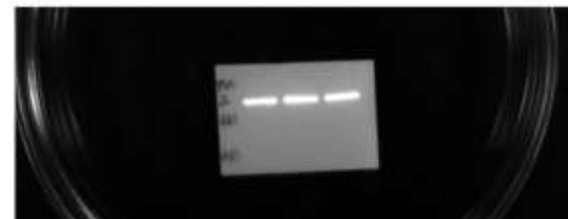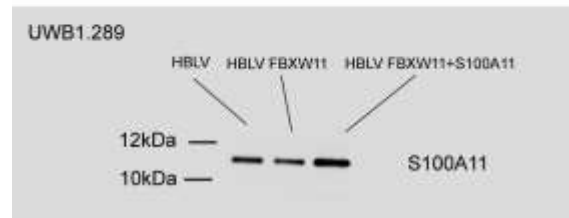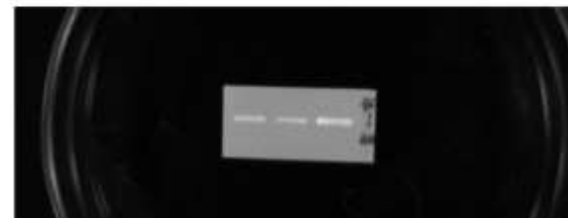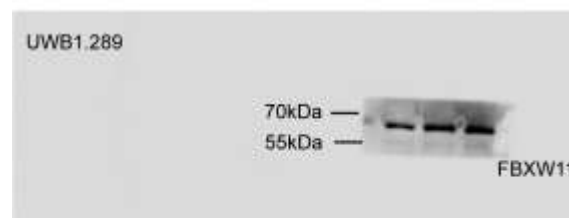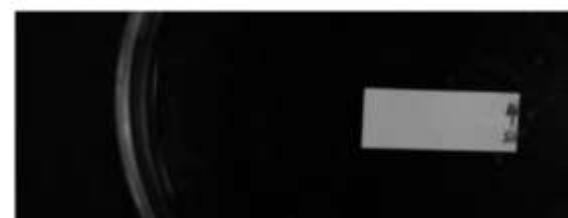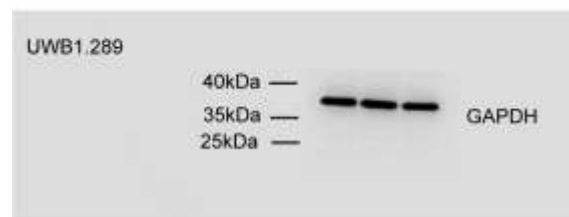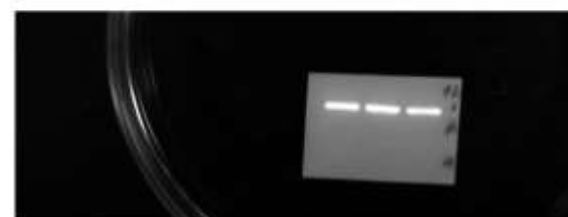

Fig.S3A

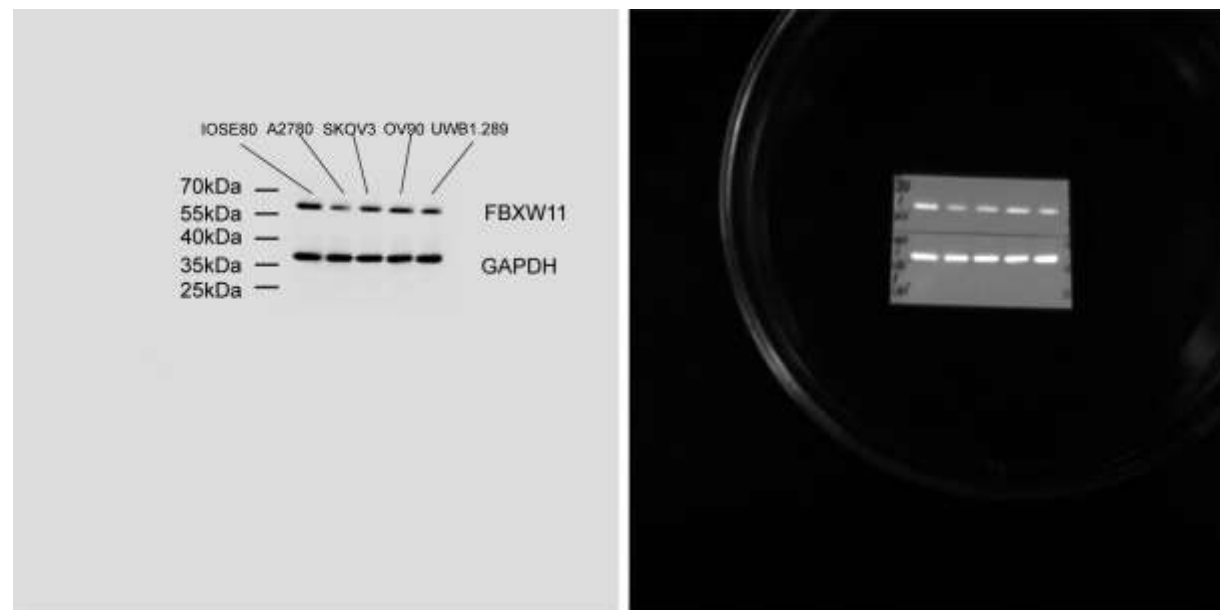

Fig.S7A

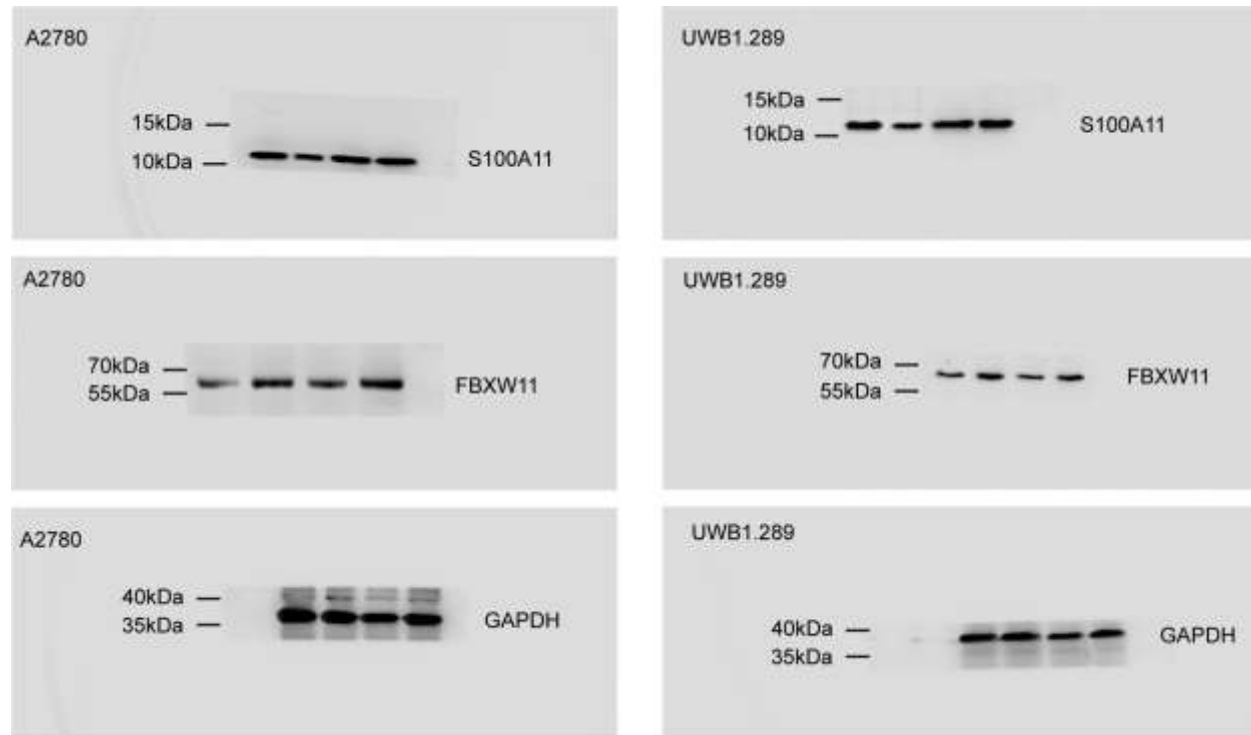

Supplement: Multimedia component 4 [file mmc4.pdf]
